# A meta-analysis of the effects of therapeutic hypothermia in adult patients with traumatic brain injury

**DOI:** 10.1186/s13054-019-2667-3

**Published:** 2019-12-05

**Authors:** Hanbing Chen, Fei Wu, Penglei Yang, Jun Shao, Qihong Chen, Ruiqiang Zheng

**Affiliations:** 1 Graduate School of Dalian Medical University; Department of Critical Care Medicine, Northern Jiangsu People’s Hospital; Clinical Medical College, Yangzhou University, No.98 Nantong West Road, Yangzhou, 225001 Jiangsu China; 2 Department of Intensive Care Unit, Affiliated Hospital of Yangzhou University, Clinical Medical College, Yangzhou University, No.368 Hanjiangzhonglu Road, Yangzhou, 225001 Jiangsu China; 3 Department of Critical Care Medicine, Northern Jiangsu People’s Hospital; Clinical Medical College, Yangzhou University, No.98 Nantong West Road, Yangzhou, 225001 Jiangsu China; 4 Department of Critical Care Medicine, Jiangdu People’s Hospital of Yangzhou, Jiangdu People’s Hospital Affiliated to Medical College of Yangzhou University, No 9 Dongfanghong Road of Jiangdu District, Yangzhou, 225001 Jiangsu China

**Keywords:** Therapeutic hypothermia, Normothermia, Traumatic brain injury, Mortality, Meta-analysis

## Abstract

**Purpose:**

Therapeutic hypothermia management remains controversial in patients with traumatic brain injury. We conducted a meta-analysis to evaluate the risks and benefits of therapeutic hypothermia management in patients with traumatic brain injury.

**Methods:**

We searched the Web of Science, PubMed, Embase, Cochrane (Central) and Clinical Trials databases from inception to January 17, 2019. Eligible studies were randomised controlled trials that investigated therapeutic hypothermia management versus normothermia management in patients with traumatic brain injury. We collected the individual data of the patients from each included study. Meta-analyses were performed for 6-month mortality, unfavourable functional outcome and pneumonia morbidity. The risk of bias was evaluated using the Cochrane Risk of Bias tool.

**Results:**

Twenty-three trials involving a total of 2796 patients were included. The randomised controlled trials with a high quality show significantly more mortality in the therapeutic hypothermia group [risk ratio (RR) 1.26, 95% confidence interval (CI) 1.04 to 1.53, *p* = 0.02]. Lower mortality in the therapeutic hypothermia group occurred when therapeutic hypothermia was received within 24 h (RR 0.83, 95% CI 0.71 to 0.96, *p* = 0.01), when hypothermia was received for treatment (RR 0.66, 95% CI 0.49 to 0.88, *p* = 0.006) or when hypothermia was combined with post-craniectomy measures (RR 0.69, 95% CI 0.48 to 1.00, *p* = 0.05). The risk of unfavourable functional outcome following therapeutic hypothermia management appeared to be significantly reduced (RR 0.78, 95% CI 0.67 to 0.91, *p* = 0.001). The meta-analysis suggested that there was a significant increase in the risk of pneumonia with therapeutic hypothermia management (RR 1.48, 95% CI 1.11 to 1.97, *p* = 0.007).

**Conclusions:**

Our meta-analysis demonstrated that therapeutic hypothermia did not reduce but might increase the mortality rate of patients with traumatic brain injury in some high-quality studies. However, traumatic brain injury patients with elevated intracranial hypertension could benefit from hypothermia in therapeutic management instead of prophylaxis when initiated within 24 h.

## Introduction

Traumatic brain injury (TBI) is a great challenge to public health; more than 50 million people suffer from TBI every year worldwide [1]. TBI can cause swelling (oedema) in the brain, can increase intracranial hypertension (ICP) and can worsen the injury. Cell death can occur minutes to hours after the injury, and the harmful effects can last for 72 h or longer [2]. Therapeutic hypothermia (TH) can reduce ICP [3] and, to some extent, play the role of a neuroprotective agent, thereby protecting the function of neurons, improving the prognosis of patients and achieving the goal of reducing mortality [4].

To date, TH in patients with TBI remains controversial. The results of a large number of animal experiments support TH management [5], and numerous studies have shown that TH can improve neurological outcomes and reduce mortality [6, 7]. However, in recent years, some studies have considered that TH, compared with the control condition, did not ameliorate outcomes among patients with severe TBI [8, 9]. Moreover, a large multicentre trial showed that TH played a negative role in the mortality rate and functional outcome [2]. From this, we can see that the TH strategies remain controversial in patients with TBI.

Systematic reviews have also reported conflicting results [10–12]. A large meta-analysis reported a benefit of TH, but this may be due to the influence of a large number of low-quality studies [12]. However, a recent meta-analysis suggested that TH could cause more mortality and poor outcomes in high-quality studies [13]. The aim of this meta-analysis is to use RCTs to update the evidence according to when and who administered TH to patients with TBI by analysing 6-month mortality rates, functional outcome, and pneumonia morbidity.

## Methods

This meta-analysis was performed according to the Preferred Reporting Items for Systematic Reviews and Meta-Analyses: the PRISMA statement [14]. The review was registered with the PROSPERO International prospective register of systematic reviews (registration number CRD42019121207).

### Eligibility criteria

All studies included in our meta-analysis met the following criteria:

1. Type of research: Clinical randomised controlled trial
2. Population: Patients with TBI
3. Intervention: TH management
4. Control: Normothermia management or fever control
5. Research outcomes:

- Primary outcomes: 6-month mortality, unfavourable functional outcome [Glasgow Outcome Scale (GOS) score 1–3: 1, death; 2, a vegetative state; 3, severe disability. Or Glasgow Outcome Scale-Extended (GOS-E) score 1–4: 1, death; 2, vegetative state; 3–4, severe disability]
- Secondary outcome: Pneumonia morbidity

### Search strategy

We searched the PubMed, Web of Science, Embase, Cochrane (Central) and Clinical Trials databases from inception to January 17, 2019, for studies discussing TH management in patients with TBI. All of the studies we included were independently screened and read by two authors. By reading the abstracts and topics, we excluded clearly unrelated literature, and by reading the full texts, we included only articles that fully met the requirements. When there was a disagreement about a study, the third author arbitrated discussions until a decision was reached. All of the included studies were limited to English articles that could be retrieved. In addition, we manually reviewed the relevant journals that were available.

### Data extraction

Data were collected using an author-created information extraction form. The two authors independently extracted the required content by screening the literature. When there was a dispute about a study, the two authors reached a consensus through discussion. If no consensus could be reached, the third author arbitrated until a final decision was reached. The data extracted from each trial included the following: first author, publication date, sample content, inclusion criteria, exclusion criteria, Glasgow Coma Score (GCS) on admission, outcome data for the GOS or GOSE, induction time, target temperature of the hypothermia group, hypothermia duration, rewarming rate, follow-up time and study results.

### Bias risk assessment

The Cochrane Collaboration’s tool for assessing the risk of bias was used. The items assessed were random sequence generation, allocation concealment, blinding of the participants and personnel, blinding of the outcome assessment, incomplete outcome data, selective reporting and other biases (Additional file 1: Figure S1). In order to quantify the quality of the articles, we performed a subgroup analysis of quality assessment according to the modified Jadad score (Additional file 13: Table S1). In addition, we also conducted a subgroup analysis with reference to the quality evaluation method of Watson et al. [13] (Additional file 14: Table S2).

### Trial sequential analysis

To prevent the constantly updated meta-analysis from increasing the risk of type I errors, we conducted a TSA that could also estimate the amount of information required for such research, thereby stopping similar research in time and preventing the waste of medical resources. We performed a one-sided TSA to summarise and analyse the data of the included studies for the functional outcome with 5% risk of type I error and 80% power.

### Statistical analysis

All statistical aspects of the meta-analysis were performed using Review Manager 5.3 software. All our outcomes comprised dichotomous data, and the pooled risk ratios and 95% confidence intervals of these data were calculated. In terms of statistical heterogeneity, a quantitative analysis was performed using the Mantel-Haenszel (MH) chi-square test and the *I*-square test; when *p* was < 0.05 for the MH chi-square test or *I*^2^ was > 50% for the *I*-square test, there was obvious heterogeneity. To evaluate the publication bias, we created funnel plot charts. In addition, we conducted a sensitivity analysis using STATA version 15.1 to determine whether any single study incurred undue weight in the analysis.

## Results

### Study selection

We present the entire search process and the reasons for excluding the ineligible studies in a flowchart in Additional file 3: Figure S3 (Additional file 2: Figure S2). Our search strategy identified 2523 studies: 555 studies were excluded due to duplicate data, 1872 studies were excluded after a review of the abstracts and titles and 88 studies were excluded after a full-text screening. The remaining 23 studies with a total of 2796 patients were included in our final analysis.

### Characteristics of the trials

We included 23 studies that compared patients with TBI in a TH group and a control group. Table 1 shows all the characteristics of the included studies. All the studies were published between 1993 and 2018, with samples ranging from 16 to 495 patients. Of these 23 trials, 22 [2, 6, 8, 15–33] compared the effect of hypothermia treatment with regular treatment or fever control (the target temperature of the TH group ranged from 32 to 35 °C). One trial [34] divided the patients into three groups: deep cooling (20 to 29 °C), mild cooling (30 to 36 °C) and a control group. We only included the data for the mild cooling group, which was comparable to the groups in our other included studies. The induction time ranged from “immediately” to 10 days, the hypothermia duration ranged from 1 to 14 days and the follow-up time ranged from 3 to 24 months. Twenty-two [2, 6, 8, 15–33] of these studies included mortality data, and 21 studies [2, 6, 8, 15–23, 25–29, 31–34] reported data on functional outcome (dichotomized GOS or GOSE).

**Table 1:** Characteristics of included studies

Study	Population		Age		GCS	Induction time	Target temperature	Hypothermia duration	Rewarming rate	Follow-up time
	HT	CON	HT	CON						
Aibiki et al. [19]	15	11	34 ± 6	38 ± 8	≤ 8	3–4 h	32–33 °C	3–4 days	1 °C/day	6 months
Andrews et al. [2]	195	192	37.4 ± 15.4	36.7 ± 14.9	3–15	3–10 days	32–35 °C	48 h	0.25 °C/h	6 months
Clifton et al. [15]	24	22	NM	NM	4–7	6 h	32–33 °C	48 h	1 °C/4 h	NM
Clifton et al. [21]	190	178	31 ± 12	32 ± 13	3–8	6 h	32.5–34.0 °C	48 h	0.5 °C/2 h	6 months
Clifton et al. [31]	52	45	26 ± 9	31 ± 11	3–8	2.5 h	33 °C	48 h	0.5 °C/2 h	6 months
Cooper et al. [8]	256	239	35 ± 13.5	34.1 ± 13.4	< 9	Rapidly	33 ± 0.5 °C	72 h	0.25 °C/h	6 months
Harris et al. [30]	12	13	38.1 ± 15	33.2 ± 20	≤ 8	24 h	33 °C	24 h	0.5 °C/3 h	NM
Hashiguchi et al. [23]	9	8	29.0 ± 14.9	39.1 ± 13.2	< 8	As soon as possible	33.5–34.5 °C	48 h	1 °C/day	6 months
Idris et al. [34]	9	13	17.3–40.5	35.0–56.1	6–7	NM	20–29 °C, 30–36 °C	24–48 h	NM	6 months
Jiang et al. [20]	43	44	Mean 42.2	Mean 40.6	≤ 8	15 h	33–35 °C	3–14 days	≤ 1 °C/h	12 months
Liu et al. [28]	43	23	NM	NM	≤ 8	2 h	33–35 °C	3 days	Passive	24 months
Maekawa et al. [32]	94	45	39 ± 19	39 ± 18	4–8	92 h	32–34 °C	≥ 72 h	< 1 °C/day	6 months
Marion et al. [17]	40	42	31 ± 12	35 ± 15	3–7	10 h	32–33 °C	24 h	Passive	12 months
Meissner et al. [24]	11	13	Median 30	Median 48	≤ 9	8 h	32–33 °C	24–48 h	NM	NM
Qiu et al. [26]	43	43	Mean 40.0	Mean 42.3	< 8	4.3 days	33–35 °C	3–5 days	Passive	24 months
Qiu et al. [29]	40	40	Mean 41.3	Mean 40.2	≤ 8	Immediately	33–35 °C	4 days	Passive	12 months
Shiozaki et al. [16]	16	17	35.3 ± 15.3	35.4 ± 12.6	≤ 8	24 h	33.5–34.5 °C	2 days	Passive	6 months
Shiozaki et al. [18]	8	8	31.4 ± 12.7	40.3 ± 23.1	≤ 8	2 h	33.5–34.5 °C	48 h	1 °C/day	6 months
Shiozaki et al. [22]	45	46	35 ± 20	42 ± 17	≤ 8	As quickly as possible	33.5–34.5 °C	48 h	1 °C/day	3 months
Smrcka et al. [27]	35	37	NM	NM	< 8	15 h	34 °C	72 h	Passive	6 months
Tang et al. [33]	30	30	42.47 ± 13.93	39.67 ± 15.26	3–8	Within 24 h	32–35 °C	48 h	0.25 °C/h	6–48 months
Zhao et al. [6]	40	41	36.9 ± 14.8	37.5 ± 15.2	3–8	3–5 h	32.5–33 °C	72 h	Passive	3 months
Zhi et al. [25]	198	198	43 ± 17	42 ± 19	≤ 8	Within 24 h	32–35 °C	1–7 days	1 °C/4 h	6 months

*HT* therapeutic hypothermia, *CON* control, *GCS* Glasgow Coma Score, *NM* not mentioned

### Mortality

Mortality was reported in 22 studies, which included a total of 2774 patients. Overall, there was no significant difference between the hypothermia group and the normothermia group (RR 0.91, 95% CI 0.80–1.03, *p* = 0.13) (Additional file 3: Figure S3). The funnel plot chart that we created showed no significant difference in publication bias between the two groups (Additional file 4: Figure S4). When we conducted a subgroup analysis according to different populations, we find that TH is more beneficial for patients in Eastern countries (RR 0.70, 95% CI 0.58–0.84, *p* = 0.0002) (Additional file 5: Figure S5).

**Fig. 1:**
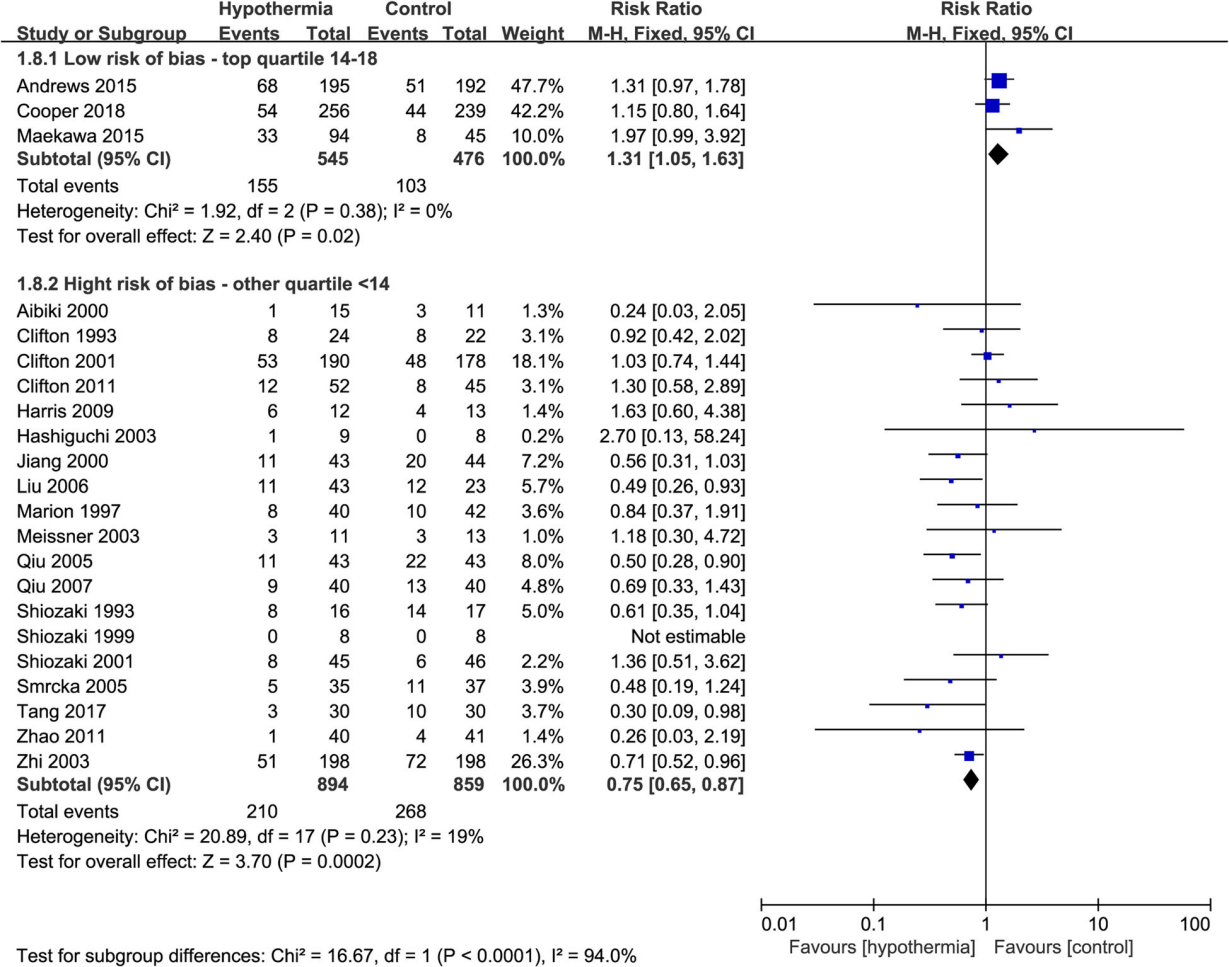
Forest plot of mortality in the low risk group or high risk group. M-H, Mantel–Haenszel method; CI, confidence interval

**Fig. 2:**
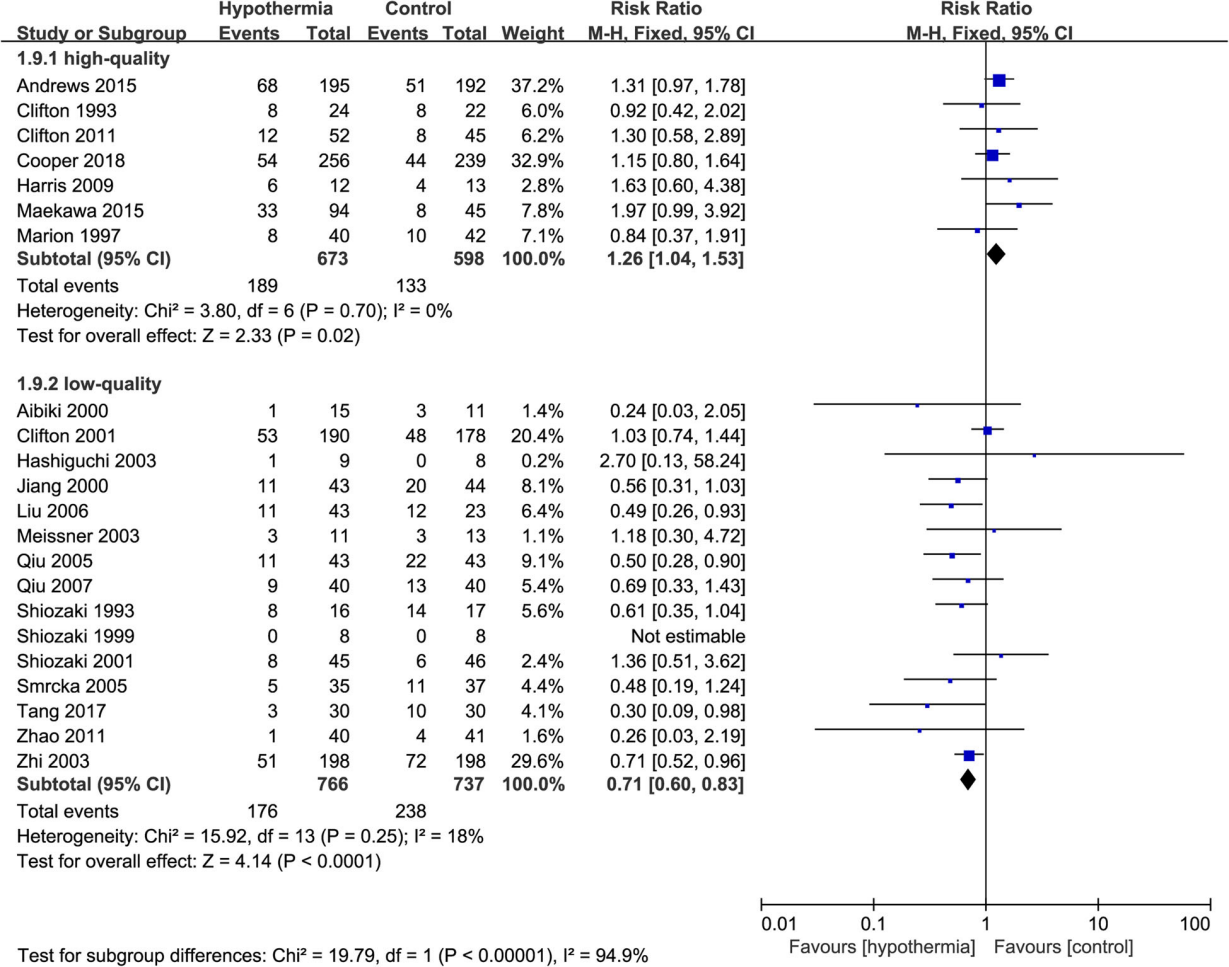
Forest plot of mortality in high-quality group or low-quality group by jaded score. M-H, Mantel–Haenszel method; CI, confidence interval

According to bias score, three studies with a low risk of bias showed a higher mortality in the TH group (RR 1.31, 95% CI 1.05–1.63, *p* = 0.02), whereas the 19 studies with high risk of bias showed a higher mortality in the control group (RR, 0.75; 95% CI, 0.65–0.87; p = 0.0002). There was possible high heterogeneity between the two subgroups (*I*^2^ = 94%) (Fig. 1). According to the modified Jadad quality score, seven studies with high quality showed a higher mortality in the TH group (RR 1.26, 95% CI 1.04–1.53, *p* = 0.02), whereas the 15 studies with low quality showed a higher mortality in the control group (RR 0.71, 95% CI 0.60–0.83, *p* < 0.0001). There was possible high heterogeneity between the two subgroups (*I*^2^ = 94.9%) (Fig. 2).

### Subgroup analyses of early TH (< 24 h) versus late TH (> 24 h)

**Fig. 3:**
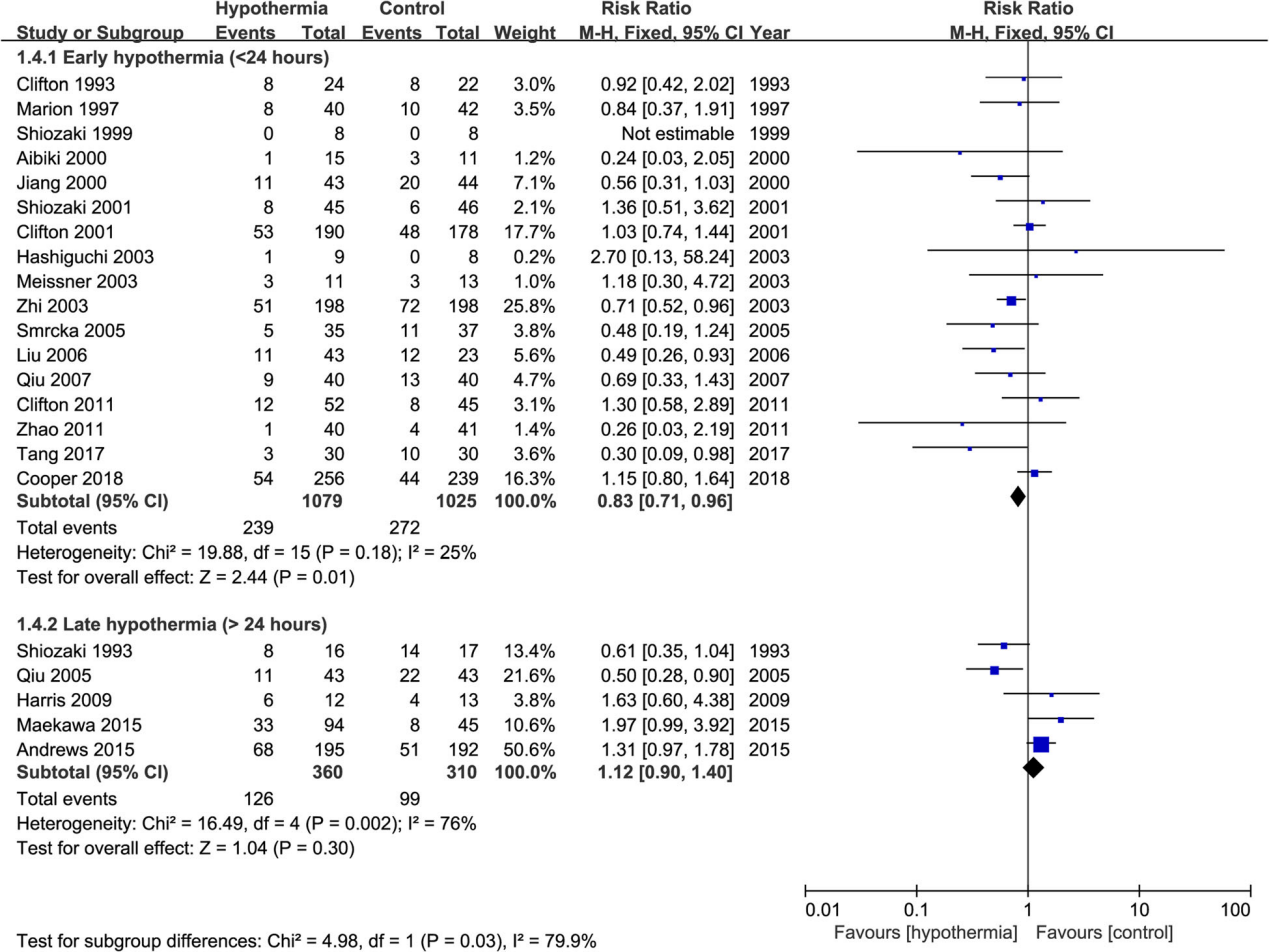
Forest plot of mortality analysed the induction time of TH. M-H, Mantel–Haenszel method; CI, confidence interval

When mortality was analysed in terms of the induction time of TH after TBI, there were significant differences between the subgroups. For the participants with early hypothermia (< 24 h), there was significantly greater mortality in the control group than in the TH group (RR 0.83, 95% CI 0.71 to 0.96, *p* = 0.01), with possible low heterogeneity (*I*^2^ = 25%). However, among those participants with TH induced ≥ 24 h after TBI, there was no significant difference in mortality between the TH and control groups (RR 1.12, 95% CI 0.90 to 1.40, *p* = 0.30) with possible substantial heterogeneity (*I*^2^ = 76%). There was also a possibility of substantial heterogeneity (*I*^2^ = 79.9%) between the two subgroups (Fig. 3).

### Subgroup analyses of TH for prevention versus treatment

**Fig. 4:**
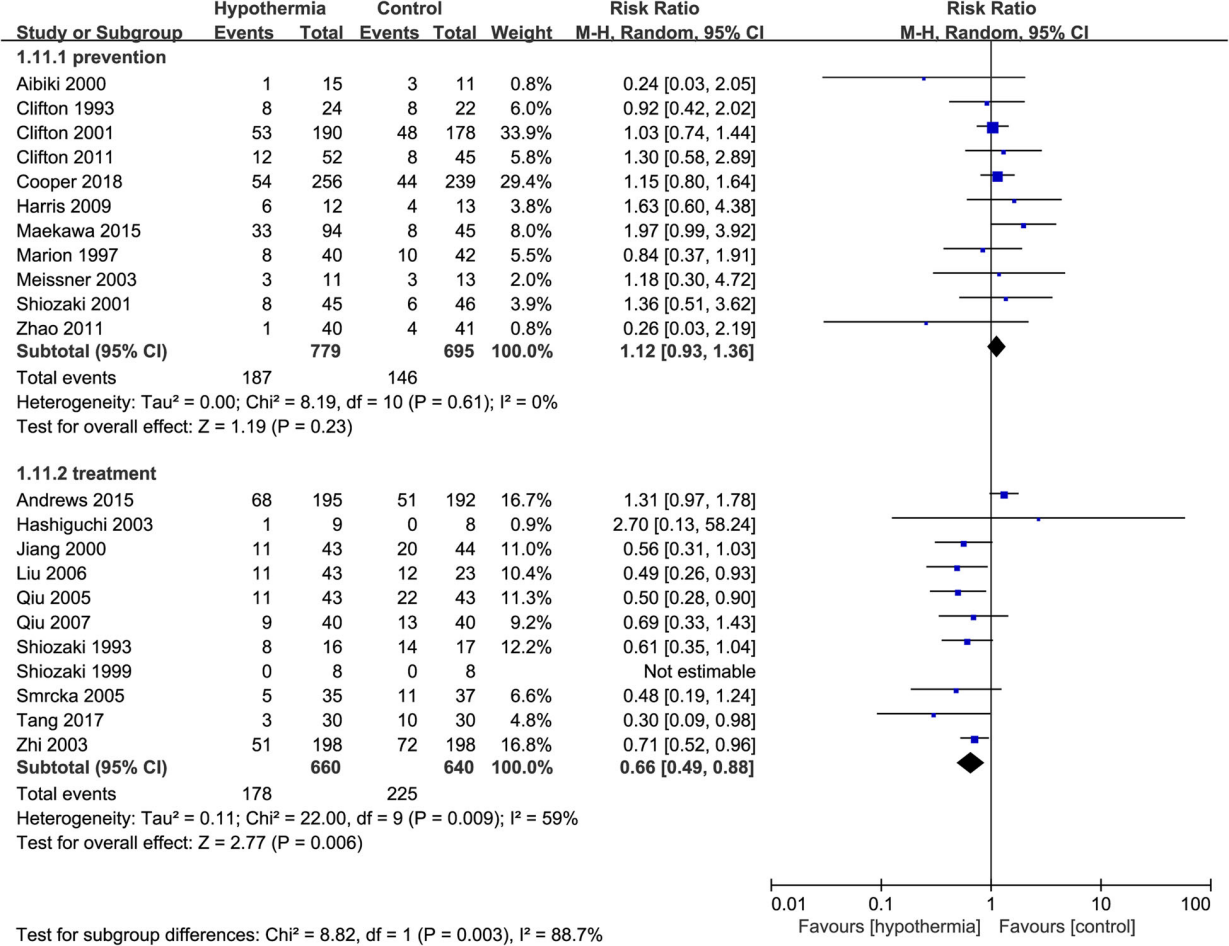
Forest plot of mortality in prevention group or treatment group. M-H, Mantel–Haenszel method; CI, confidence interval

When mortality was analysed in terms of hypothermia for prevention or treatment, among those who received TH for treatment there was significantly greater mortality in the control group than in the TH group (RR 0.66, 95% CI 0.49 to 0.88, *p* = 0.006), with possible moderate heterogeneity (*I*^2^ = 59%). However, among those who received TH for prevention, there was no significant difference in mortality between the TH and control groups (RR 1.12, 95% CI 0.93 to 1.36, *p* = 0.23) with possible low heterogeneity (*I*^2^ = 0%). There was also a possibility substantial heterogeneity (*I*^2^ = 88.7%) between the subgroups (Fig. 4).

### Subgroup analyses using TH in post-craniectomy versus non-craniectomy

**Fig. 5:**
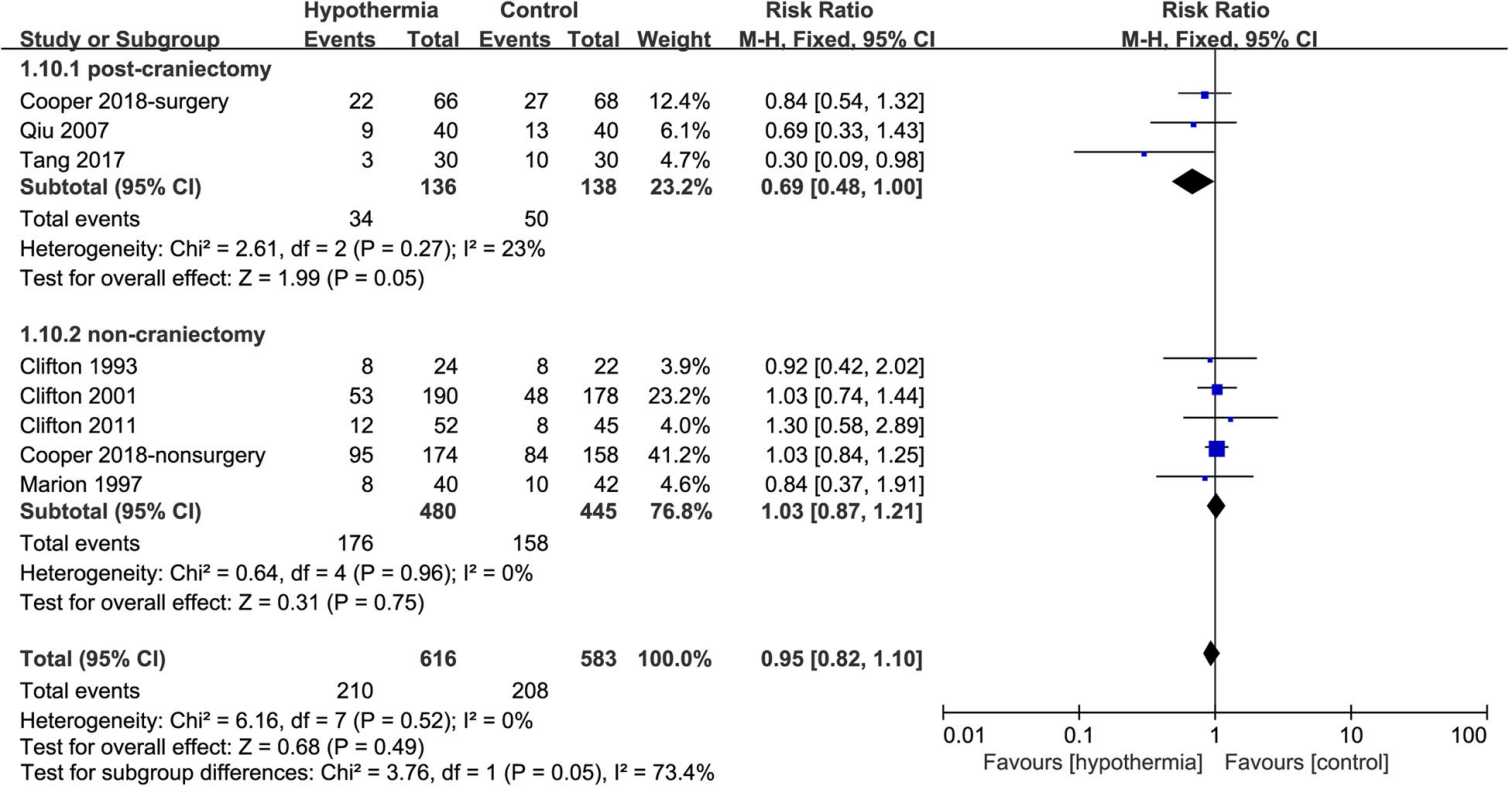
Forest plot of mortality in post-craniectomy group or non-craniectomy group. M-H, Mantel–Haenszel method; CI, confidence interval

When mortality was analysed in terms of using TH in post-craniectomy or non-craniectomy patients, among those participants who received TH in post-craniectomy, there was lower mortality in the TH group than in the control group (RR 0.69, 95% CI 0.48 to 1.00, *p* = 0.05), with possible low heterogeneity (*I*^2 =^ 23%). However, among those participants who received TH without a craniectomy for TBI, there was no significant difference in mortality between the TH and control groups (RR 1.03, 95% CI 0.87 to 1.21, *p* = 0.75) with possible low heterogeneity (*I*^2^ = 0%). There was also a possibility of substantial heterogeneity (*I*^2^ = 73.4%) between the two subgroups (Fig. 5).

### Unfavourable functional outcome

**Fig. 6:**
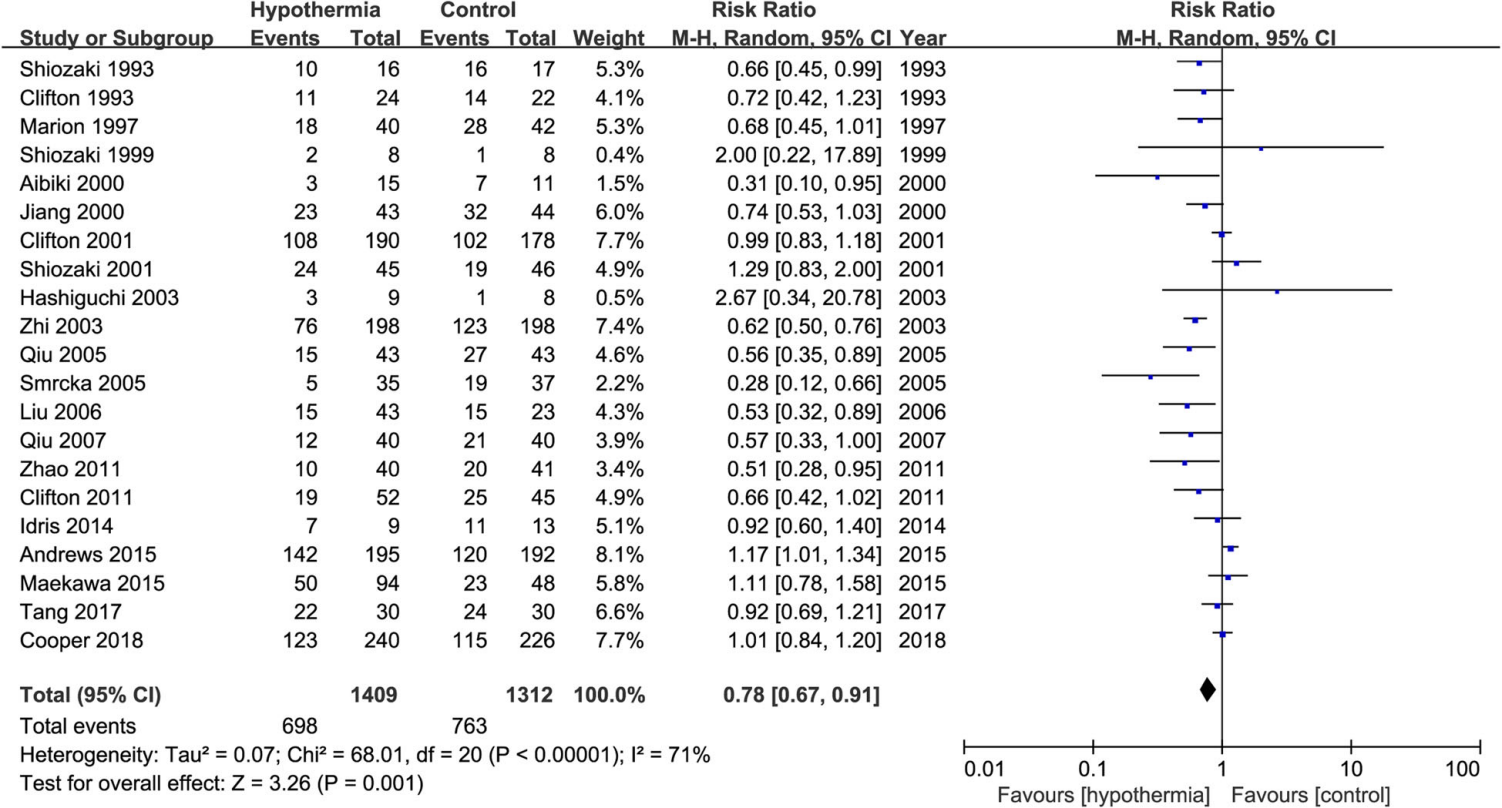
Risk ratio of unfavourable functional outcome in the TH group versus control group. M-H, Mantel–Haenszel method; CI, confidence interval

The impact of TH on functional outcome was evaluated in 21 trials that included a total of 2721 patients. A total of 1409 patients were assigned to the TH group, 698 of whom had an unfavourable functional outcome. A total of 1312 patients were assigned to the control group; 763 of these patients had an unfavourable functional outcome. The funnel plot chart that we created showed no significant difference in publication bias between the two groups (Additional file 6: Figure S6). The results showed that the risk of an unfavourable functional outcome was significantly reduced in the TH group versus the control group (RR 0.78, 95% CI 0.67 to 0.91, *p* = 0.001) (Fig. 6). The TSA showed that the required information size (RIS) for such studies was 3864 patients. Furthermore, the *Z*-curve crossed both the traditional boundary and the TSA line but did not reach the RIS line, which shows that the current number of trials may have reached a positive conclusion regarding the neurological prognosis (Additional file 7: Figure S7).

### Secondary outcomes

**Fig. 7:**
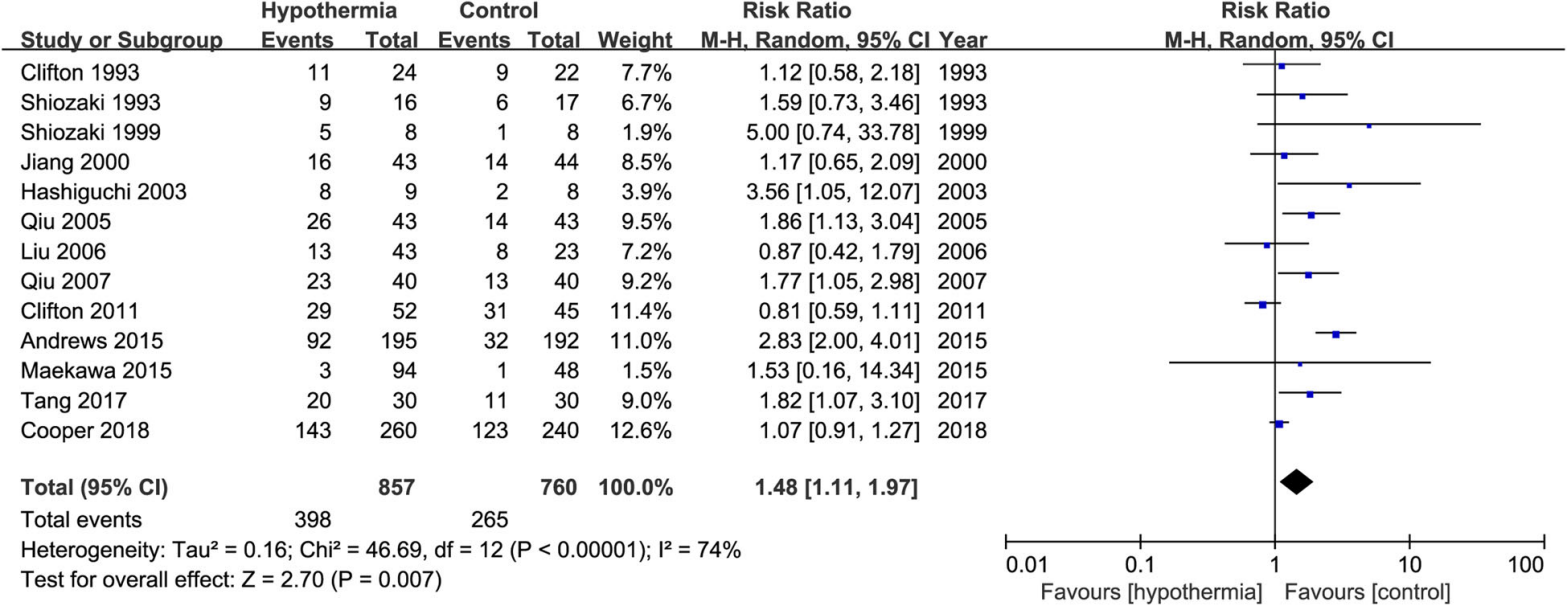
Risk ratio of pneumonia in the TH group versus control group. M-H, Mantel–Haenszel method; CI, confidence interval

Thirteen studies [2, 8, 15, 16, 18, 20, 23, 26, 28, 29, 31–33] reported pneumonia as an outcome. Overall, there was a significant increase in the risk of pneumonia with TH management (RR 1.48, 95% CI 1.11 to 1.97, *p* = 0.007). There was possible substantial heterogeneity (*I*^2^ = 74%) between the TH group and the control group (Fig. 7).

### Sensitivity analysis

We systematically and qualitatively analysed the sensitivity across the included studies to determine the influence of individual trials on the results. We did not detect a significant impact from any single study and confirmed the direction of the results (Additional file 8: Figure S8, Additional file 9: Figure S9 and Additional file 10: Figure S10).

## Discussion

TH management remains controversial for patients with TBI [11, 35]. Despite extensive research, there is no high-quality evidence that hypothermia is beneficial to TBI patients, as is to cardiac arrest [36]. Similar to a recent meta-analysis, our meta-analysis demonstrated that TH could cause more mortality in the subgroup of high-quality studies. And TH initiated within 24 h could reduce mortality in patients with TBI [13]. Furthermore, we also find TBI patients benefit from TH when hypothermia is used for therapy instead of prophylaxis. Additionally, post-craniectomy TBI patients may benefit more from TH than patients who have not received a craniectomy. In terms of functional outcome, our meta-analysis is consistent with previous meta-results [2, 12]. Patients with TBI can show improved neurological outcomes with TH within 72 h of injury [8].

Some RCTs suggest that elevated ICP is associated with worsening outcomes in patients with TBI [37, 38]. Elevated ICP may result in decreased cerebral perfusion pressure and cerebral blood flow, which may further lead to hypoxic-ischaemic brain damage [39]. Many studies have shown that TH can play a role in neuroprotection in many ways, mainly because hypothermia can reduce ICP, reduce the brain metabolic rate, reduce the blood flow in the brain, change the release of neurotransmitters and maintain the function of the blood-brain barrier [40, 41]. Moreover, TH can reduce the inflammatory response and biochemical cascade that is activated early after TBI [42], thereby limiting secondary brain injury [43, 44]. A study by Roman et al. showed that TH can improve the functional prognosis of GOS (4–5) by reducing ICP [7]. Our study also found that hypothermia can improve the patient’s functional outcomes.

However, some studies have shown that hypothermia sometimes plays an adverse role. Several recent multicentre large RCT studies have shown that TH not only failed to reduce patient 6-month mortality but may also be harmful to patients with lesser damage [2, 8, 9, 32]. Long-term hypothermia is considered a form of immunosuppression that increases the infection rate of pneumonia and sepsis [45]. In addition, it has been reported in the literature that hypothermia can cause propofol infusion syndrome because propofol can reduce liver metabolism; this may be an important cause of fatal symptoms at low temperatures [46]. It has also been reported that low temperatures can affect the metabolism of certain drugs, including muscle relaxants such as atracurium, which may further affect 6-month mortality [47]. The CRASH study, a large multicentre trial, also found a lower 6-month survival rate in the hypothermia group and a higher 2-week mortality rate in patients treated with methylprednisolone [48]. It has been shown that the use of steroidal antipyretics may also be one of the important factors that influence mortality. The results of our subgroup analysis also showed that high-quality studies suggested that TH can cause an increase in mortality.

We observed smaller studies may note some “benefits” from hypothermia while the more structured large RCTs have failed; we believe it may be because the smaller studies are mostly with small sample size and single-centre. Through the sample size-bias curve, we found that the sample size and the bias score showed a significant positive correlation after the abnormal point was removed (Additional file 11: Figure S11), that is, as the sample size increased, the bias gradually decreased. However, the study of Zhi et al. has a large sample size but a low bias score, and different to the results of other studies with large sample size, we suspect that it may invite bias into the results. The column chart about research centres and bias also support our conjecture (Additional file 12: Figure S12). But since all the included studies meet the inclusion criteria for our meta-analysis, there is no reason to remove any RCT study, which is a limitation of our research. So we recommend more large multicentre RCTs to continue this research.

Why is there controversy regarding TH management for patients with TBI? First, it seems that the induction time of TH is the key point. Our meta-analysis found that both survival rates and functional outcomes will benefit if TH is administered within 24 h after TBI. In animal experiments, the most obvious link between intracranial temperature changes and nerve injury occurred within the first 24 h [49, 50]. When TH is applied within 24 h after TBI, it may be possible to control the increase of ICP earlier, thereby reducing intracranial nerve injury and improving the functional prognosis of patients. There are data indicating that hypothermia may regulate both the JNK signalling cascade via XIAP and the preconditioning pathways that activate caspases. Thus, hypothermia mediates TNFR1 responses via early activation of the JNK signalling pathway and caspase-3, leading to endogenous neuroprotective events [51]. Recent studies by Watson et al. also supported early hypothermia in patients with TBI [13]. At present, there is no consensus regarding when TH management should be used after TBI. After the occurrence of TBI, the severity of numerous destructive biochemical cascades plays a decisive role in the survival of nerve cells [43]. TH is an effective protective mechanism to inhibit these reactions. We believe that TH management within 24 h is conducive to maximally limiting the infinite expansion of these cascades in a short period of time, thereby avoiding risks, and when the TH time is later (more than 24 h), patients may have more serious damage, and ICP may be more difficult to control; such destructive reactions have been irreversible.

A subgroup analysis of TH for prevention or treatment suggests that TH may be more effective in reducing mortality when used for therapeutic purposes. We believe that the hypothermia applied in patients with TBI after cerebral oedema, increased intracranial pressure or craniotomy is defined as a therapeutic effect, and the application of hypothermia as soon as possible without relevant complications is a preventive effect. These two concepts have not been clearly defined internationally, and we need to recognise the subjectivity of this subgroup analysis. Moreover, the subgroup analysis of post-craniotomy showed that TH after surgery had a tendency to reduce mortality. Previous studies have reported that the use of mild hypothermia as a preventive application of neuroprotective agents has also failed; prophylactic hypothermia is not recommended to improve final outcomes [52]. Clifton et al. also found that, compared with diffuse brain injury, TH may play a better effect in those with surgically evacuated haematomas [31]. It has also been confirmed at the experimental level that intra-ischaemic hypothermia after haematoma removal is associated with improved outcomes [53]. Through the analysis of the above two subgroups, we hypothesise that we can use hypothermia as a treatment for TBI patients while evacuating the haematoma and after cranietomy, which can effectively reduce the mortality rate.

We need to discuss some of the limitations of our work. First, although we performed a comprehensive database search and a manual search and made a funnel plot, and the funnel plot had symmetry, we did not search the grey literature or contact authors to confirm whether there were any unpublished studies. Therefore, we still cannot rule out the existence of a publication bias. Second, the inclusion criteria for each group of trials included in this study were not completely consistent, which may have led to heterogeneity in the observations. The forest plot shows that the difference in weight is relatively large, which may affect the final result to some extent. Finally, we found substantial heterogeneity in some of the outcomes. We tried to reduce clinical and methodological heterogeneity through different subgroup analyses; however, some analyses did not have an obvious effect, and the heterogeneity was still high. Therefore, we used a random effects model instead of a fixed effects model to address the observed heterogeneity. Despite such differences, our sensitivity analysis identified no outlier studies, hinting that our results were relatively reliable.

## Conclusions

Our meta-analysis demonstrated that TH did not reduce but might increase the mortality rate of patients with TBI in some high-quality studies. However, TBI patients with elevated ICP could benefit from hypothermia in therapy instead of in prophylaxis when initiated within 24 h, which may require further research to confirm.

## Supplementary information

**Additional file 1: Figure S1.** Risk of bias summary review authors’ judgement about each risk of bias item for each included study. Red, high risk; green, low risk; yellow, unclear

**Additional file 2: Figure S2.** Study flow diagram detailing the literature search. GOS = Glasgow Outcome Scale

**Additional file 3: Figure S3.** Risk ratio of mortality in the TH group versus control group. M-H = Mantel–Haenszel method, CI = confidence interval

**Additional file 4: Figure S4.** The funnel plot for mortality. SE = standard error, RR = risk ratio (equivalently, relative risk)

**Additional file 5: Figure S5.** Forest plot of mortality analyzed different populations. M-H = Mantel–Haenszel method, CI = confidence interval

**Additional file 6: Figure S6.** The funnel plot for unfavorable functional outcome. SE = standard error, RR = risk ratio (equivalently, relative risk)

**Additional file 7: Figure S7.** TSA for unfavorable functional outcome in randomized controlled trials: one-sided boundary, incidence of 58.2% in the control arm, incidence of 49.5% in the intervention arm, α of 5%, and power of 80% were set. RIS  =  required information size.

**Additional file 8: Figure S8.** Sensitivity analysis of mortality. CI = confidence interval

**Additional file 9: Figure S9.** Sensitivity analysis of unfavorable functional outcome. CI = confidence interval

**Additional file 10: Figure S10.** Sensitivity analysis of pneumonia. CI = confidence interval

**Additional file 11: Figure S11.** Bias curve between sample size and bias score. correlation coefficient r = 0.678, coefficient of determination R^2^ = 0.46. *p* = 0.001

**Additional file 12: Figure S12.** Column chart about research centres and bias. t = 2.3, *p* = 0.032

**Additional file 13: Table S1.** Modified Jadad scoring for the included RCTs (*n* = 22)

**Additional file 14: Table S2.** The risk of bias of included RCTs

## Data Availability

All data generated or analysed during this study are included in this published article.

## Abbreviations

RIS: Required information size
GCS: Glasgow Coma Score
GOS: Glasgow Outcome Scale
GOS-E: Glasgow Outcome Scale-Extended
ICP: Intracranial hypertension
TBI: Traumatic brain injury
TH: Therapeutic hypothermia
TSA: Trial sequential analysis
