# Supplementary figures and images for: A meta-analysis of the effects of therapeutic hypothermia in adult patients with traumatic brain injury
Source: Crit Care. 2019 Dec 5;23:396. doi: 10.1186/s13054-019-2667-3 (PMC6896404; doi:10.1186/s13054-019-2667-3)

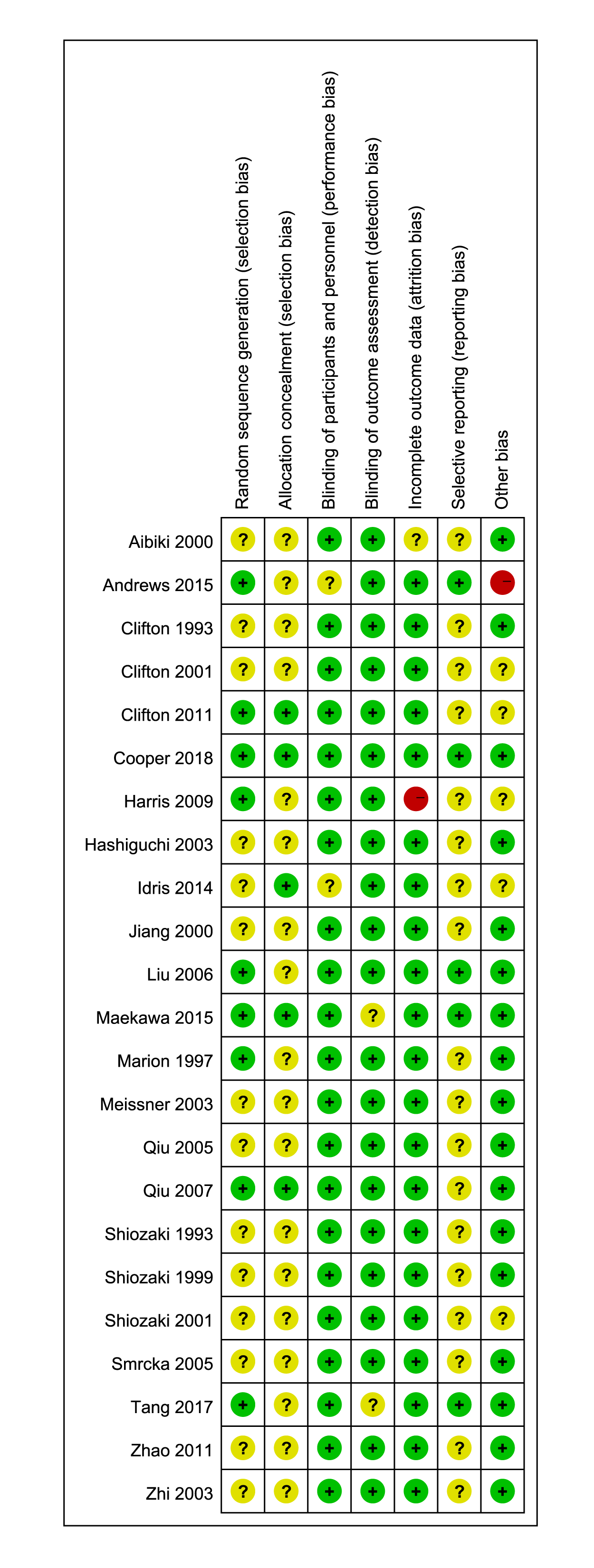

Supplement: Supplementary file 1 — Additional file 1: Figure S1. Risk of bias summary review authors’ judgement about each risk of bias item for each included study. Red, high risk; green, low risk; yellow, unclear [file 13054_2019_2667_MOESM1_ESM.tif]

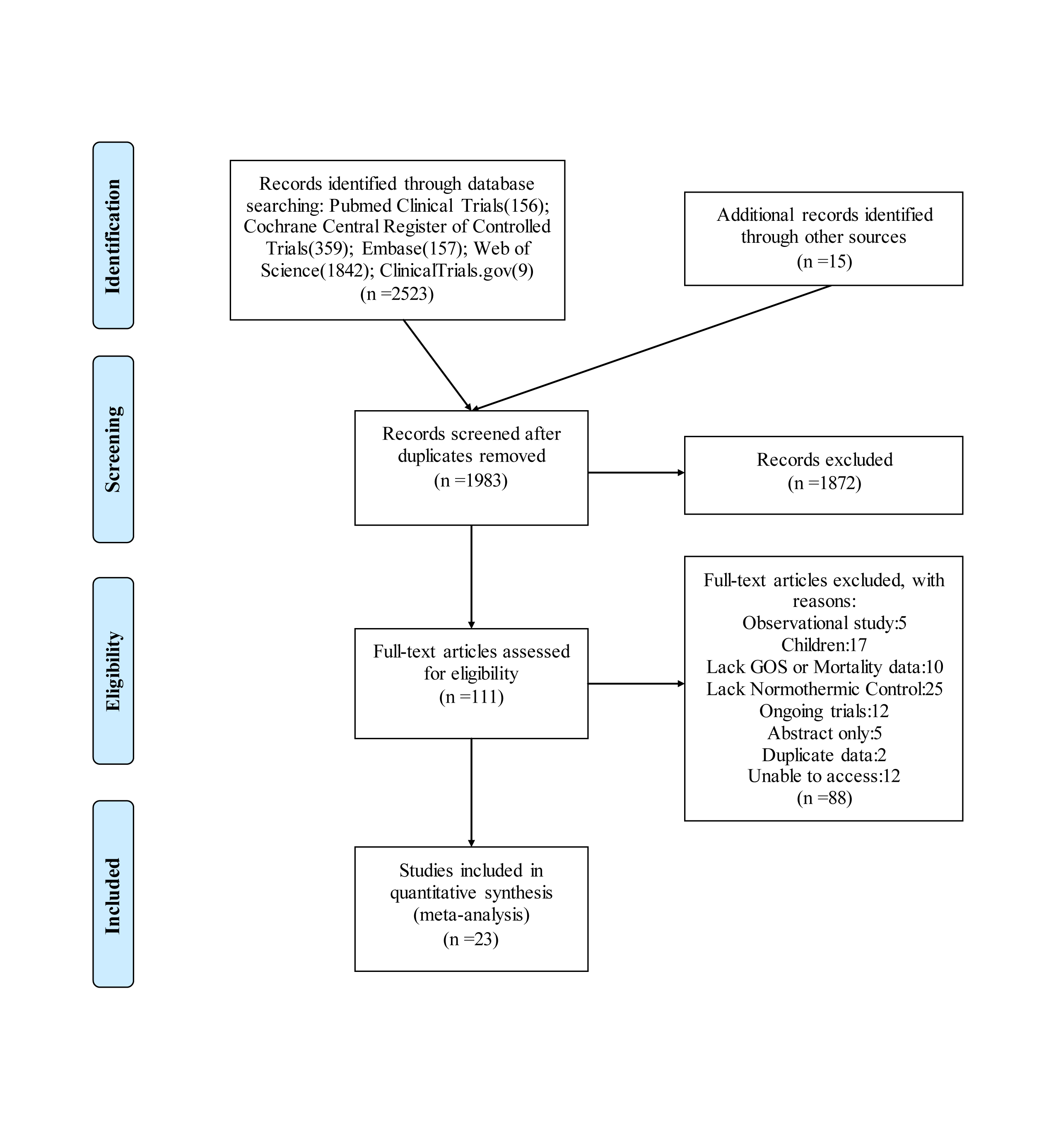

Supplement: Supplementary file 2 — Additional file 2: Figure S2. Study flow diagram detailing the literature search. GOS = Glasgow Outcome Scale [file 13054_2019_2667_MOESM2_ESM.tif]

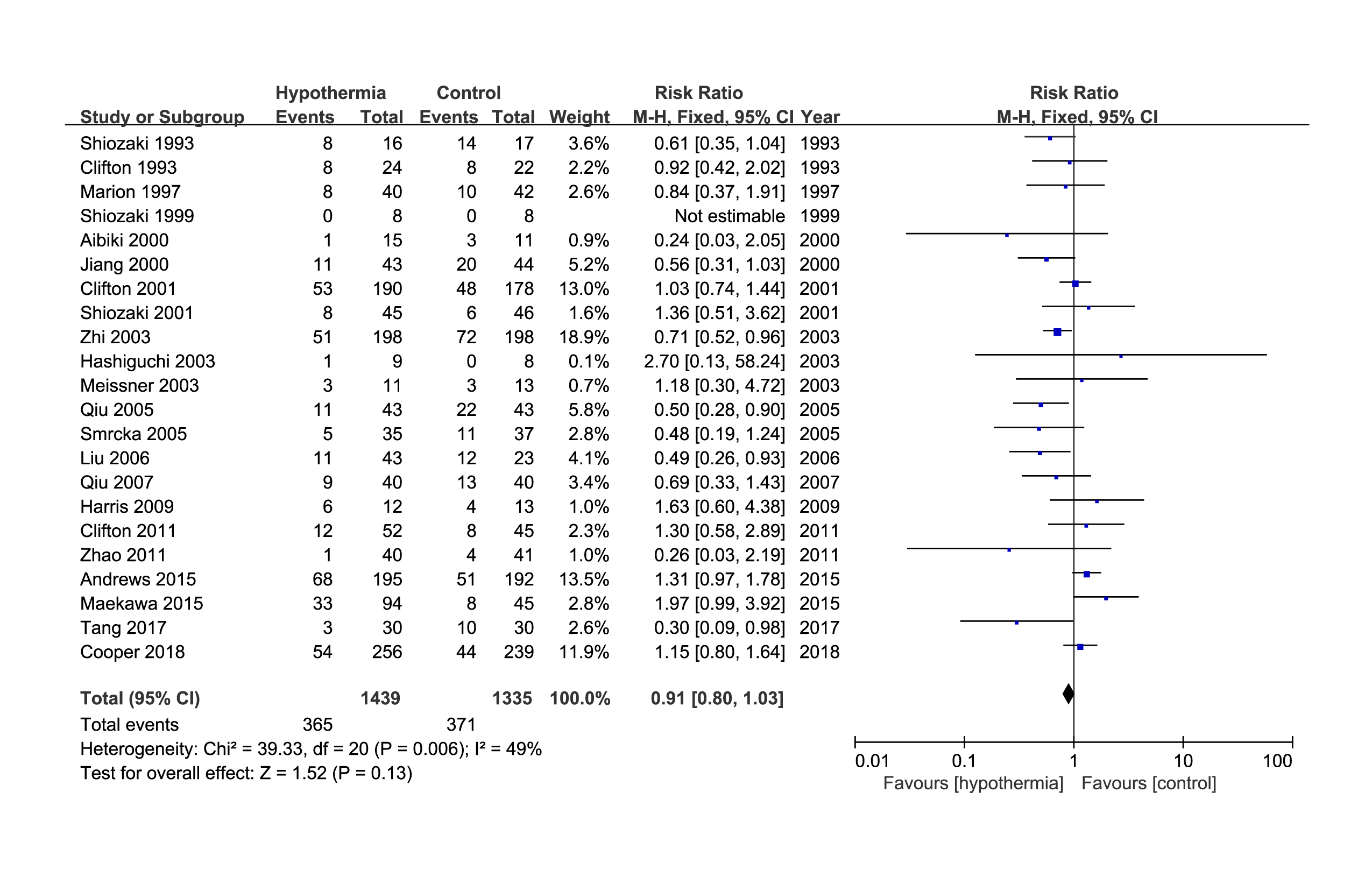

Supplement: Supplementary file 3 — Additional file 3: Figure S3. Risk ratio of mortality in the TH group versus control group. M-H = Mantel–Haenszel method, CI = confidence interval [file 13054_2019_2667_MOESM3_ESM.tif]

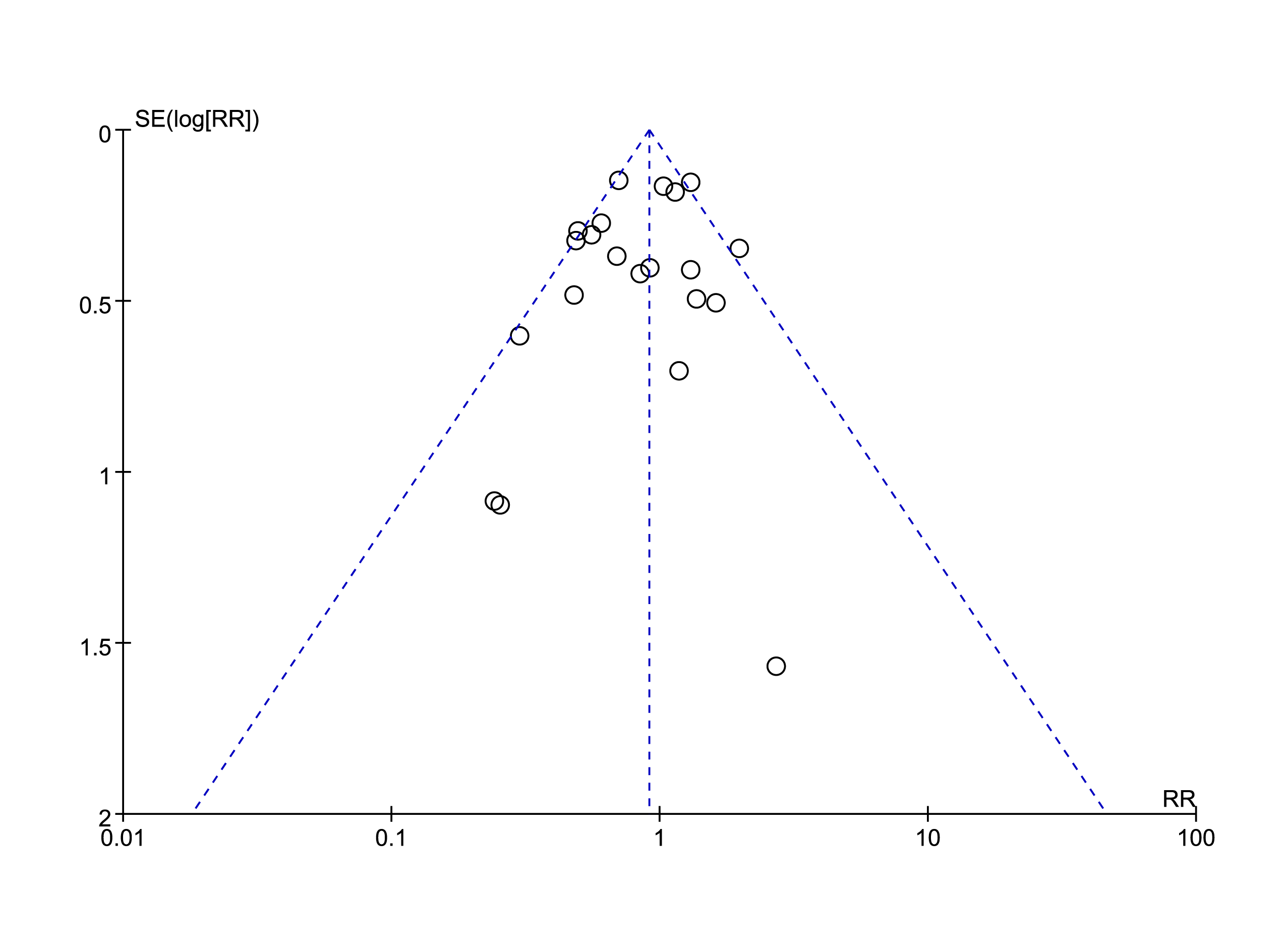

Supplement: Supplementary file 4 — Additional file 4: Figure S4. The funnel plot for mortality. SE = standard error, RR = risk ratio (equivalently, relative risk) [file 13054_2019_2667_MOESM4_ESM.tif]

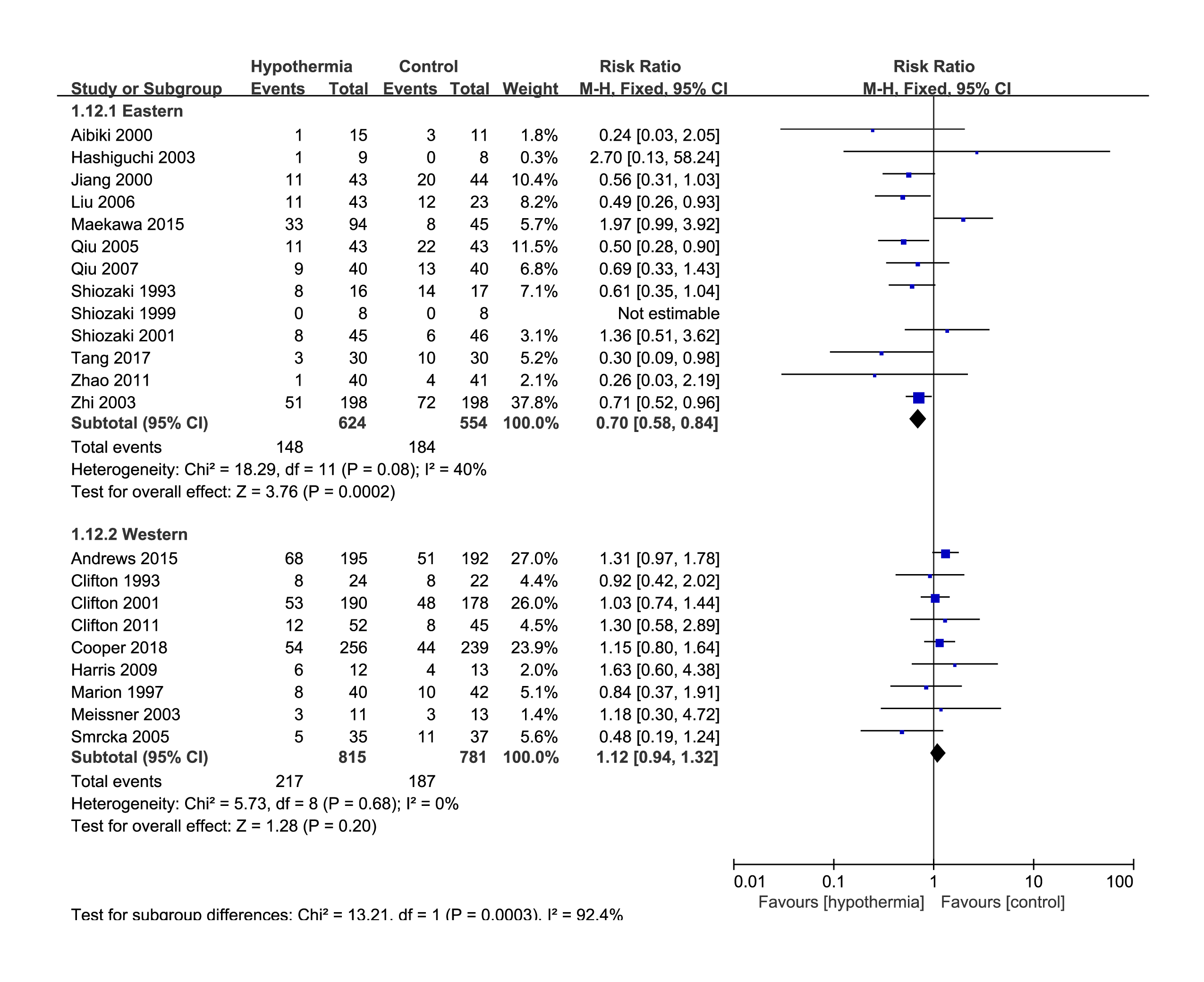

Supplement: Supplementary file 5 — Additional file 5: Figure S5. Forest plot of mortality analyzed different populations. M-H = Mantel–Haenszel method, CI = confidence interval [file 13054_2019_2667_MOESM5_ESM.tif]

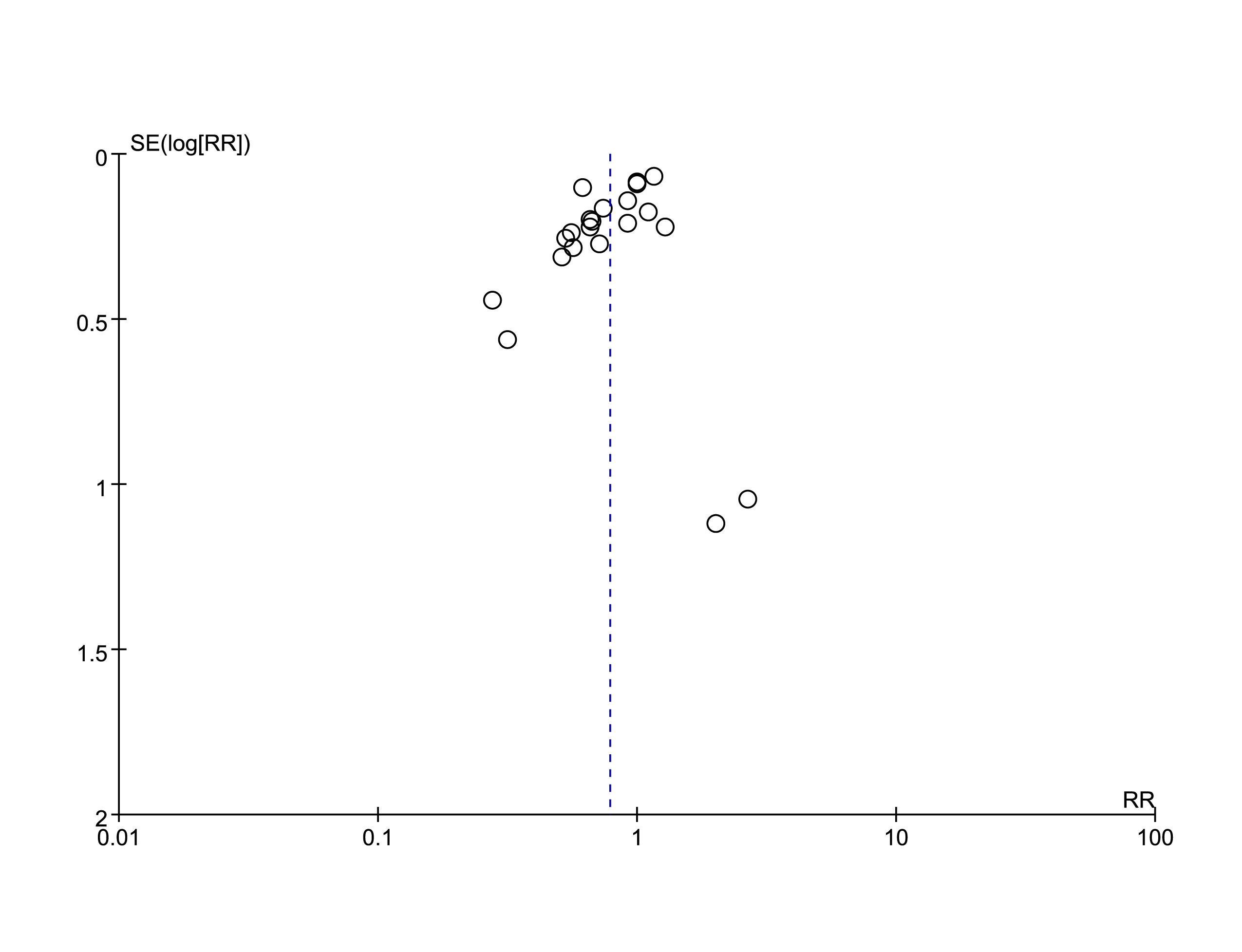

Supplement: Supplementary file 6 — Additional file 6: Figure S6. The funnel plot for unfavorable functional outcome. SE = standard error, RR = risk ratio (equivalently, relative risk) [file 13054_2019_2667_MOESM6_ESM.tif]

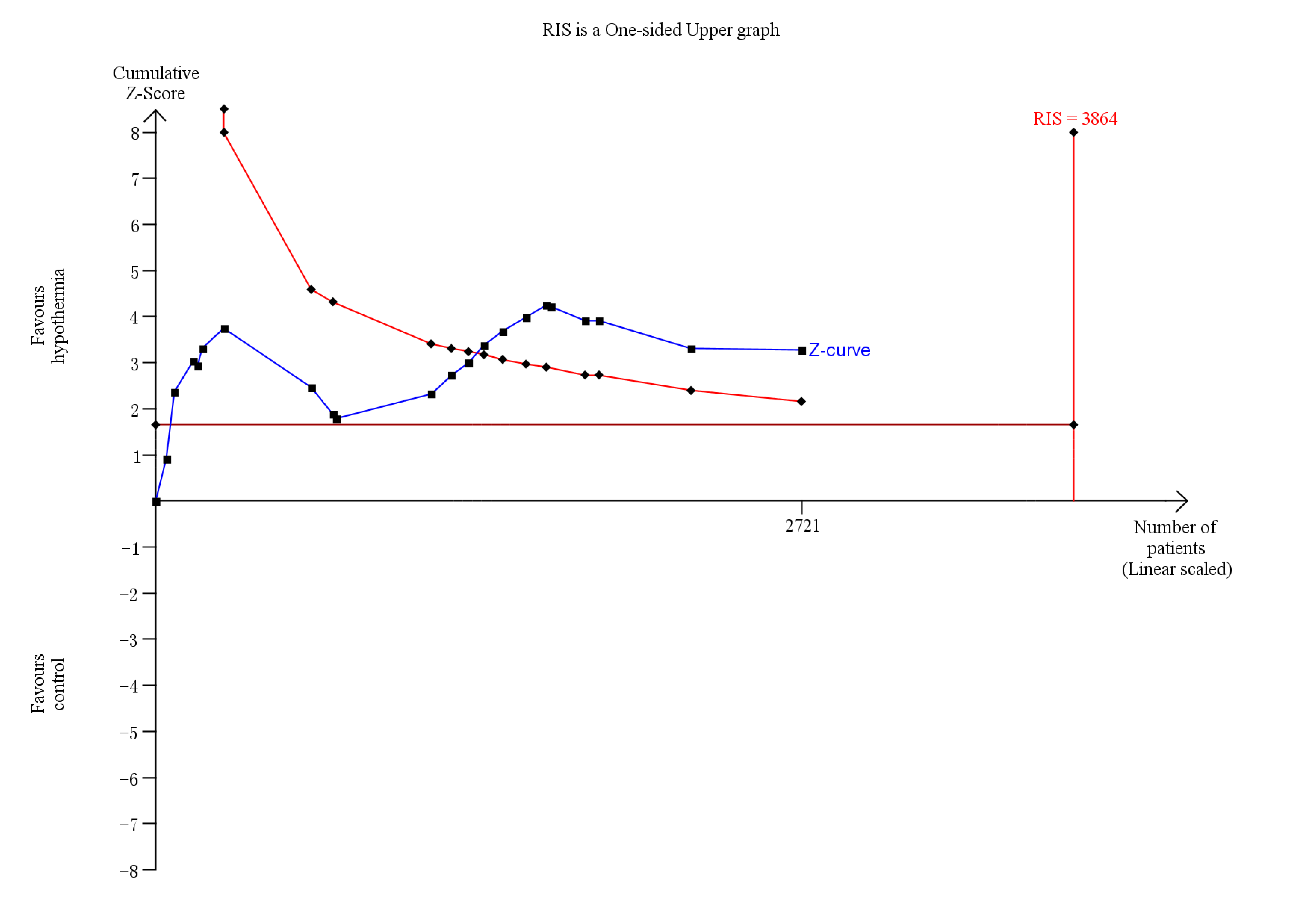

Supplement: Supplementary file 7 — Additional file 7: Figure S7. TSA for unfavorable functional outcome in randomized controlled trials: one-sided boundary, incidence of 58.2% in the control arm, incidence of 49.5% in the intervention arm, α of 5%, and power of 80% were set. RIS = required information size. [file 13054_2019_2667_MOESM7_ESM.tif]

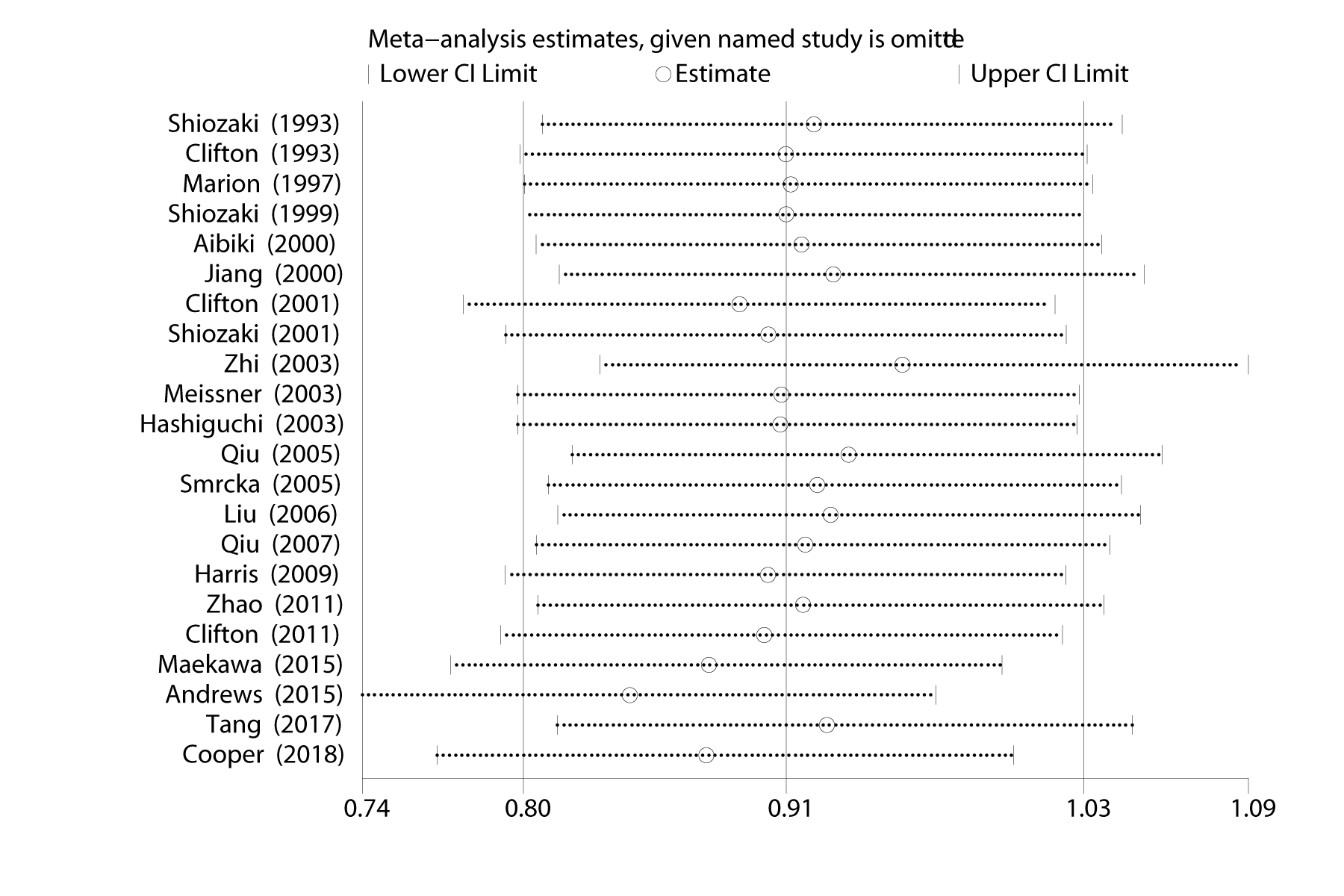

Supplement: Supplementary file 8 — Additional file 8: Figure S8. Sensitivity analysis of mortality. CI = confidence interval [file 13054_2019_2667_MOESM8_ESM.tif]

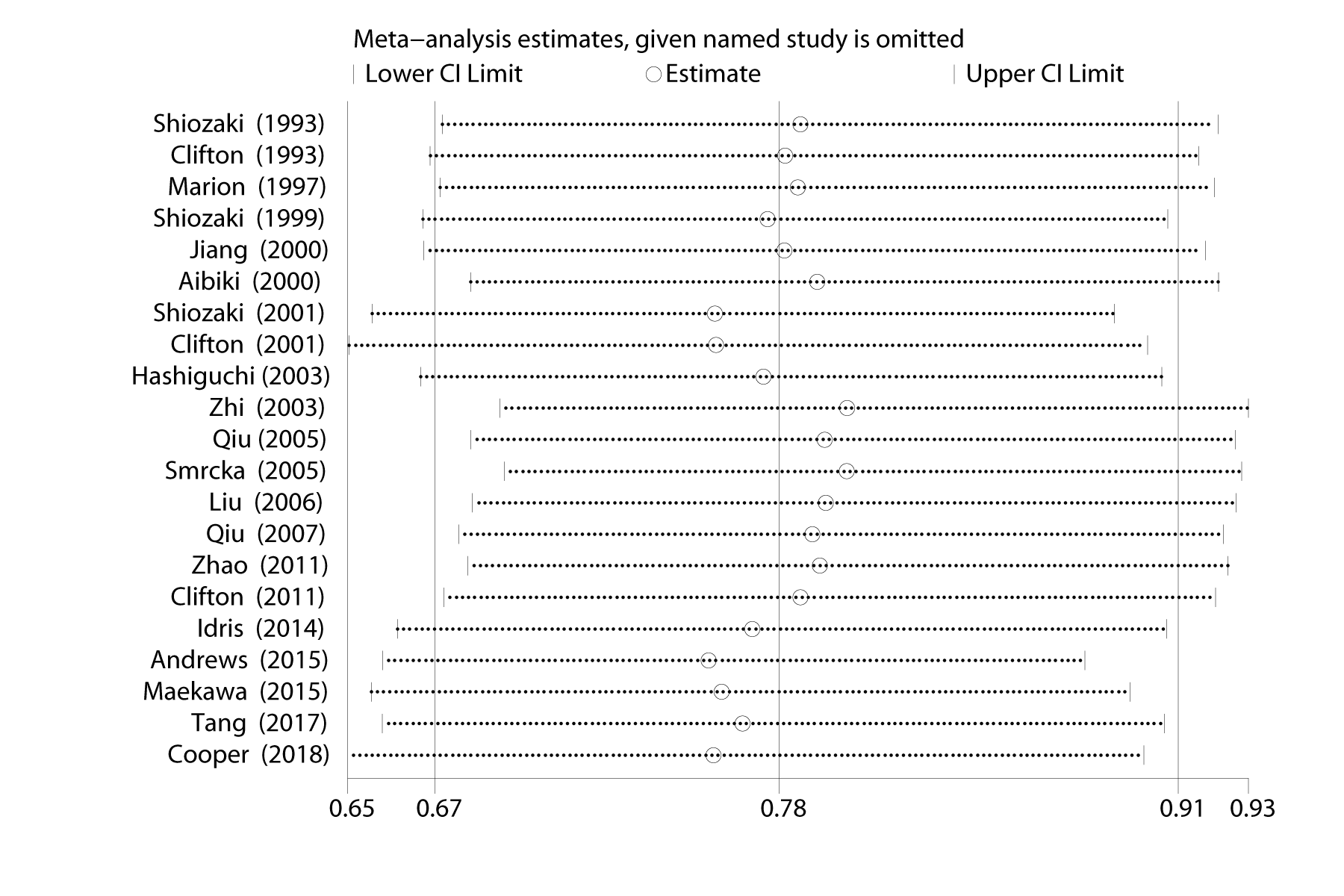

Supplement: Supplementary file 9 — Additional file 9: Figure S9. Sensitivity analysis of unfavorable functional outcome. CI = confidence interval [file 13054_2019_2667_MOESM9_ESM.tif]

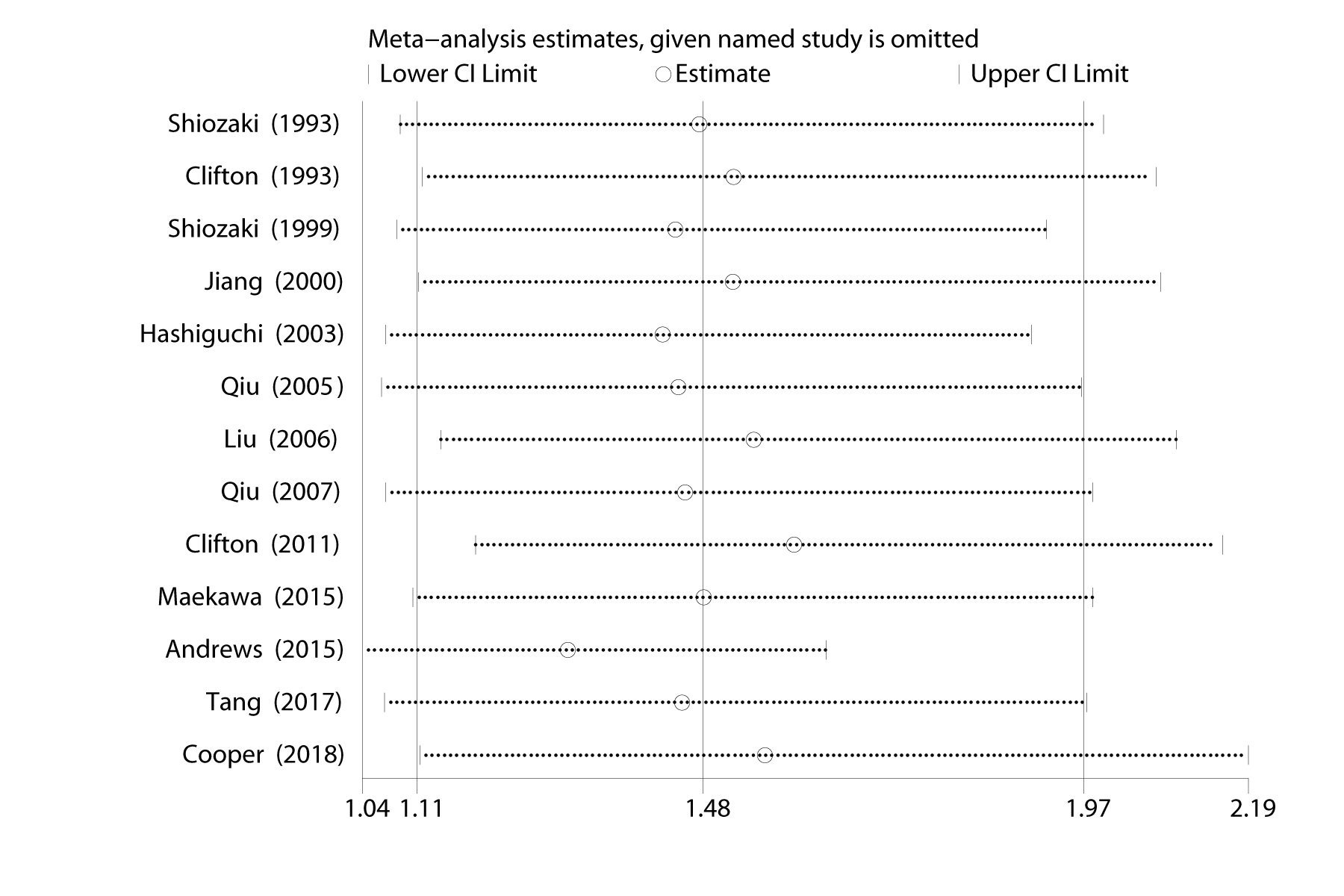

Supplement: Supplementary file 10 — Additional file 10: Figure S10. Sensitivity analysis of pneumonia. CI = confidence interval [file 13054_2019_2667_MOESM10_ESM.tif]

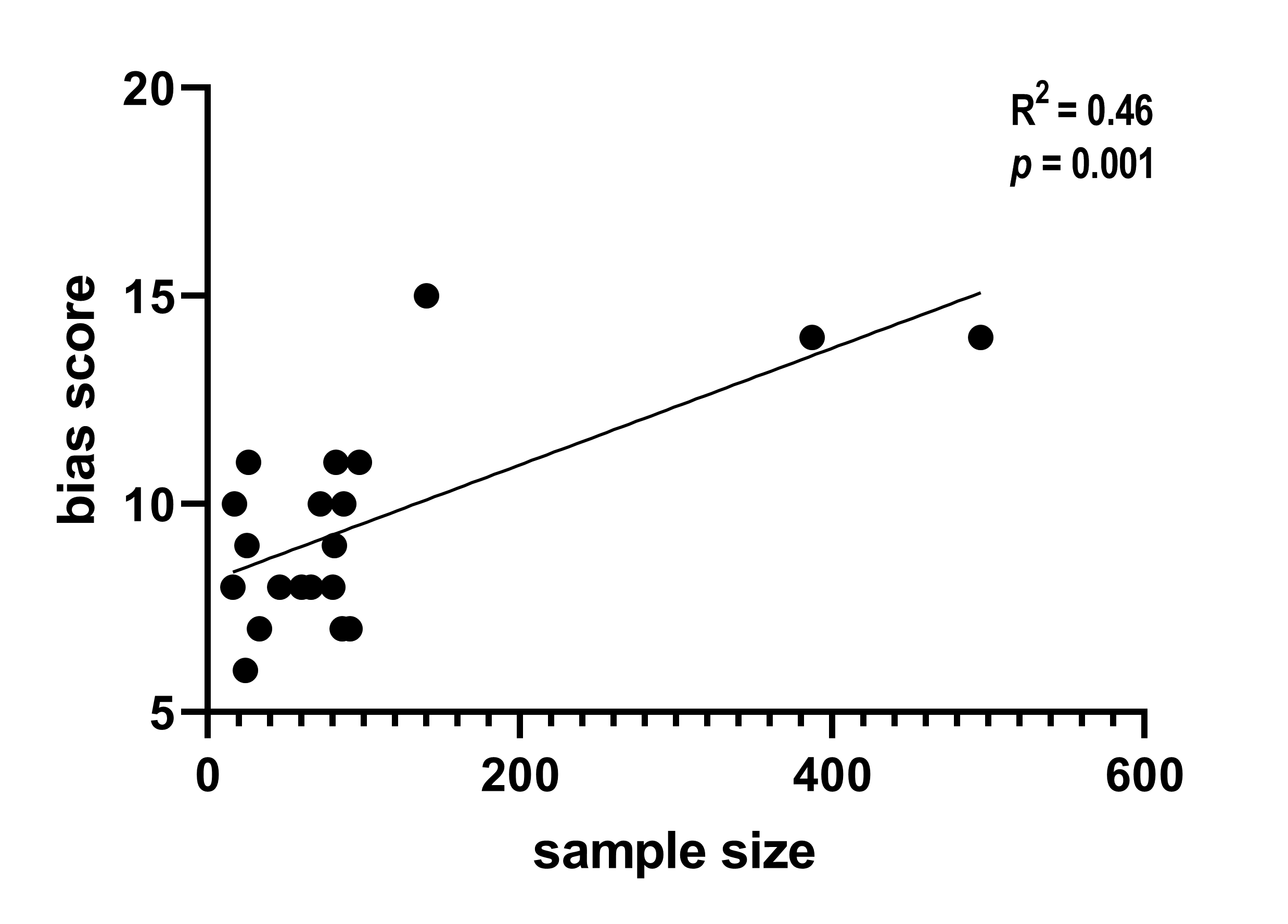

Supplement: Supplementary file 11 — Additional file 11: Figure S11. Bias curve between sample size and bias score. correlation coefficient r = 0.678, coefficient of determination R2 = 0.46. p = 0.001 [file 13054_2019_2667_MOESM11_ESM.tif]

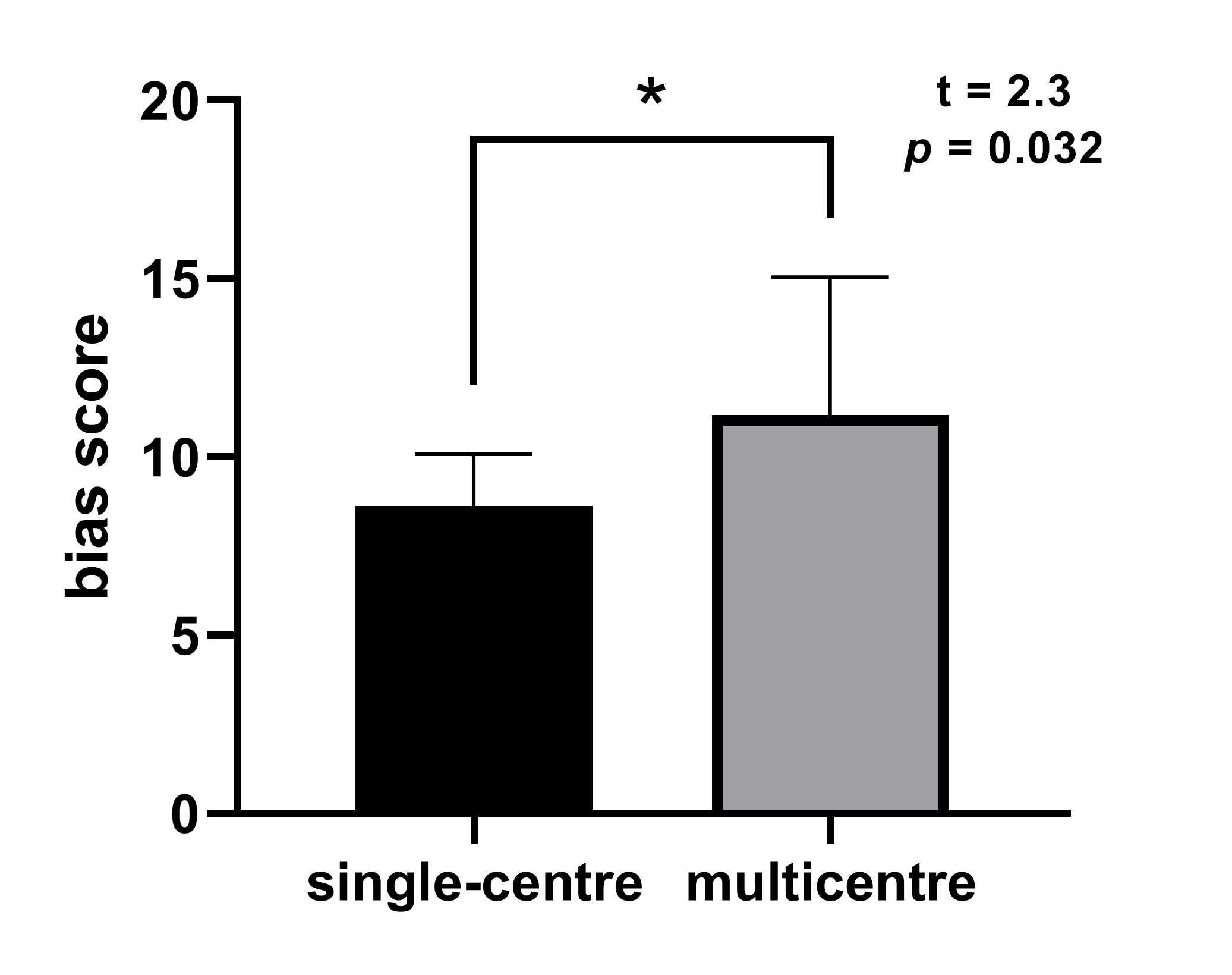

Supplement: Supplementary file 12 — Additional file 12: Figure S12. Column chart about research centres and bias. t = 2.3, p = 0.032 [file 13054_2019_2667_MOESM12_ESM.tif]
