# Supplementary material for: A meta-analysis of the effects of therapeutic hypothermia in adult patients with traumatic brain injury
Source: Crit Care. 2019 Dec 5;23:396. doi: 10.1186/s13054-019-2667-3 (PMC6896404; doi:10.1186/s13054-019-2667-3)
Supplement: Supplementary file 13 — Additional file 13: Table S1. Modified Jadad scoring for the included RCTs (n = 22) [file 13054_2019_2667_MOESM13_ESM.docx]

| Table S1: Modified Jadad scoring for the included RCTs (n = 22) | | | | | |
| --- | --- | --- | --- | --- | --- |
| **study** | **Randomization** | **Blinding** | **withdrawal** | **Total modified Jadad score** | **High quality or Low quality** |
| Clifton 1993 | 2 | 0 | 1 | 3 | H |
| Shiozaki 1993 | 1 | 0 | 1 | 2 | L |
| Marion 1997 | 2 | 0 | 1 | 3 | H |
| Shiozaki 1999 | 1 | 0 | 1 | 2 | L |
| Aibiki2000 | 0 | 0 | 0 | 0 | L |
| Jiang 2000 | 0 | 0 | 0 | 0 | L |
| Clifton 2001 | 0 | 0 | 0 | 0 | L |
| Shiozaki2001 | 0 | 0 | 1 | 1 | L |
| Hashigushi 2003 | 1 | 0 | 1 | 2 | L |
| Meissner2003 | 0 | 0 | 1 | 1 | L |
| Zhi 2003 | 0 | 0 | 1 | 1 | L |
| Qiu 2005 | 0 | 0 | 1 | 1 | L |
| Smrcka 2005 | 0 | 0 | 1 | 1 | L |
| Liu 2006 | 2 | 0 | 0 | 2 | L |
| Qiu 2007 | 1 | 0 | 1 | 2 | L |
| Harris2009 | 2 | 0 | 1 | 3 | H |
| Clifton 2011 | 2 | 0 | 1 | 3 | H |
| Zhao 2011 | 0 | 0 | 0 | 0 | L |
| Andrews 2015 | 2 | 0 | 1 | 3 | H |
| Maekawa 2015 | 2 | 0 | 1 | 3 | H |
| Tang 2017 | 2 | 0 | 0 | 2 | L |
| Cooper2018 | 2 | 0 | 1 | 3 | H |
| H, High quality; L, Low quality | | | | | |

**Randomization:** 0-point indicates randomization was not stated;

1-point indicates randomization was mentioned;

2-point indicates randomization was appropriate.

**Blinding:** not possible due to the nature of the intervention.

**Withdrawal:** 1-point indicates all patients entered into the trial are accounted for at the end of the trial;

0-point indicates someone entered into the trial are not accounted for at the end of the trial.

**High quality or Low quality:** High quality: 3 points**;** Low quality: 0, 1 or 2 points.
