# Supplementary material for: A meta-analysis of the effects of therapeutic hypothermia in adult patients with traumatic brain injury
Source: Crit Care. 2019 Dec 5;23:396. doi: 10.1186/s13054-019-2667-3 (PMC6896404; doi:10.1186/s13054-019-2667-3)
Supplement: Supplementary file 14 — Additional file 14: Table S2. The risk of bias of included RCTs [file 13054_2019_2667_MOESM14_ESM.docx]

| Study; First author & year of publication | Shiozaki 1993 | Clifton 1993 | Marion 1997 | Shiozaki 1999 | Aibiki 2000 | Jiang 2000 | Clifton 2001 |
| --- | --- | --- | --- | --- | --- | --- | --- |
| Has the control group been managed to normothermia? | Y | Y | Y | Y | Y | Y | Y |
| Yes=1 No/NS=0 | 1 | 1 | 1 | 1 | 1 | 1 | 1 |
| Were the control group actively warmed on admission if hypothermic? | NS | NS | N | NS | NS | NS | NS |
| Yes=0 No=2 NS=1 | 1 | 1 | 2 | 1 | 1 | 1 | 1 |
| Has the treatment arm received barbiturates in addition to therapeutic hypothermia? | Y | N | NS | Y | N | N | Y |
| Yes=0 No=2 NS=1 | 0 | 2 | 1 | 0 | 2 | 2 | 0 |
| Are there significant differences between the treatment and control sample populations? | N | N | N | N | N | N | N |
| Yes=0 No=1 NS=0 | 1 | 1 | 1 | 1 | 1 | 1 | 1 |
| Has the ‘standard’ treatment that the control group received been clearly outlined? | N | Y | Y | Y | Y | N | Y |
| Yes=1 No=0 | 0 | 1 | 1 | 1 | 1 | 0 | 1 |
| Adequacy of randomisation technique? | Unclear | Unclear | Adequate | Unclear | Unclear | Unclear | Unclear |
| Adequate=1 Inadequate/unclear=0 | 0 | 0 | 1 | 0 | 0 | 0 | 0 |
| Blinding of investigators | NS | NS | NS | N | NS | NS | NS |
| Yes=1 No/NS=0 | 0 | 0 | 0 | 0 | 0 | 0 | 0 |
| Blinding of participants | NS | NS | NS | NS | NS | NS | NS |
| Yes=1 No/NS=0 | 0 | 0 | 0 | 0 | 0 | 0 | 0 |
| Blinding of outcome assessor | NS | Y | NS | NS | Y | Y | Y |
| Yes=1 No/NS=0 | 0 | 1 | 0 | 0 | 1 | 1 | 1 |
| Blinding of data analysis | NS | NS | NS | NS | NS | NS | N |
| Yes=1 No/NS=0 | 0 | 0 | 0 | 0 | 0 | 0 | 0 |
| Intention to treat analysis | NS but confirmed | NS | NS but confirmed | NS but confirmed | NS | NS | Stated but not  confirmed |
| Yes=1; Not stated but confirmed on study assessment=1; Stated but not confirmed on study assessment=0; No/NS=0 | 1 | 0 | 1 | 1 | 0 | 0 | 0 |
| Completion of follow-up (at 6 months if possible) | 100% | 98% | 100% | 100% | 100% | 100% | 93.9% |
| 100%=2; >=95%=1; <95%=0; No/NS=0 | 2 | 1 | 2 | 2 | 2 | 2 | 0 |
| Reasons given when patients were excluded from enrolment, allocation follow-up or analysis? | Y | N | Y | Y | N | N | Y |
| Yes=1 No=0 | 1 | 0 | 1 | 1 | 0 | 0 | 1 |
| Has the first author published 2+ RCTs that have been included in this review? | Y | Y | Y | Y | N | N | Y |
| Yes=0 No=2 | 0 | 0 | 0 | 0 | 2 | 2 | 0 |
|  |  |  |  |  |  |  |  |
| TOTAL SCORE | **7** | **8** | **11** | **8** | **11** | **10** | **6** |

Continuation table

| Study; First author & year of publication | Shiozaki 2001 | Zhi 2003 | Meissner 2003 | Hashiguchi 2003 | Qiu 2005 | Smrcka 2005 | Liu 2006 | Qiu 2007 |
| --- | --- | --- | --- | --- | --- | --- | --- | --- |
| Has the control group been managed to normothermia? | Y | Y | Y | Y | Y | Y | Y | Y |
| Yes=1 No/NS=0 | 1 | 1 | 1 | 1 | 1 | 1 | 1 | 1 |
| Were the control group actively warmed on admission if hypothermic? | NS | Y | Y | N | NS | NS | NS | NS |
| Yes=0 No=2 NS=1 | 1 | 0 | 0 | 2 | 1 | 1 | 1 | 1 |
| Has the treatment arm received barbiturates in addition to therapeutic hypothermia? | Y | NS | NS | Y | N | NS | NS | N |
| Yes=0 No=2 NS=1 | 0 | 1 | 1 | 0 | 2 | 1 | 1 | 2 |
| Are there significant differences between the treatment and control sample populations? | N | Y | Y | Y | NS | N | N | N |
| Yes=0 No=1 NS=0 | 1 | 0 | 0 | 0 | 0 | 1 | 1 | 1 |
| Has the ‘standard’ treatment that the control group received been clearly outlined? | Y | Y | Y | Y | Y | N | N | Y |
| Yes=1 No=0 | 1 | 1 | 1 | 1 | 1 | 0 | 0 | 1 |
| Adequacy of randomisation technique? | Unclear | Unclear | Unclear | Unclear | Unclear | Unclear | Unclear | Adequate |
| Adequate=1 Inadequate/unclear=0 | 0 | 0 | 0 | 0 | 0 | 0 | 0 | 1 |
| Blinding of investigators | N | NS | NS | NS | NS | NS | NS | Y |
| Yes=1 No/NS=0 | 0 | 0 | 0 | 0 | 0 | 0 | 0 | 1 |
| Blinding of participants | NS | NS | NS | NS | NS | NS | NS | NS |
| Yes=1 No/NS=0 | 0 | 0 | 0 | 0 | 0 | 0 | 0 | 0 |
| Blinding of outcome assessor | NS | NS | NS | NS | NS | NS | NS | NS |
| Yes=1 No/NS=0 | 0 | 0 | 0 | 0 | 0 | 0 | 0 | 0 |
| Blinding of data analysis | NS | NS | NS | NS | NS | NS | NS | NS |
| Yes=1 No/NS=0 | 0 | 0 | 0 | 0 | 0 | 0 | 0 | 0 |
| Intention to treat analysis | NS | NS but confirmed | NS | NS but confirmed | NS but confirmed | NS but confirmed | NS | NS |
| Yes=1; Not stated but confirmed on study assessment=1; Stated but not confirmed on study assessment=0; No/NS=0 | 0 | 1 | 0 | 1 | 1 | 1 | 0 | 0 |
| Completion of follow-up (at 6 months if possible) | 100% | 100% | 85.7% | 100% | 89% | 100% | 100% | NS |
| 100%=2; >=95%=1; <95%=0; No/NS=0 | 2 | 2 | 0 | 2 | 0 | 2 | 2 | 0 |
| Reasons given when patients were excluded from enrolment, allocation follow-up or analysis? | Y | N | Y | Y | Y | Y | N | N |
| Yes=1 No=0 | 1 | 0 | 1 | 1 | 1 | 1 | 0 | 0 |
| Has the first author published 2+ RCTs that have been included in this review? | Y | N | N | N | Y | N | N | Y |
| Yes=0 No=2 | 0 | 2 | 2 | 2 | 0 | 2 | 2 | 0 |
|  |  |  |  |  |  |  |  |  |
| TOTAL SCORE | **7** | **8** | **6** | **10** | **7** | **10** | **8** | **8** |

Continuation table

| Study; First author & year of publication | Harris 2009 | Zhao 2011 | Clifton 2011 | Idris 2014 | Maekawa 2015 | Andrews 2015 | Tang 2017 | Cooper 2018 |
| --- | --- | --- | --- | --- | --- | --- | --- | --- |
| Has the control group been managed to normothermia? | Y | Y | Y | NS | Yes | Yes | Yes | Y |
| Yes=1 No/NS=0 | 1 | 1 | 1 | 0 | 1 | 1 | 1 | 1 |
| Were the control group actively warmed on admission if hypothermic? | NS | NS | N | NS | NS | NS | NS | NS |
| Yes=0 No=2 NS=1 | 1 | 1 | 2 | 1 | 1 | 1 | 1 | 1 |
| Has the treatment arm received barbiturates in addition to therapeutic hypothermia? | N | NS | NS | NS | N | N | NS | N |
| Yes=0 No=2 NS=1 | 1 | 1 | 1 | 1 | 2 | 2 | 1 | 2 |
| Are there significant differences between the treatment and control sample populations? | N | Y | Y | Y | N | N | N | N |
| Yes=0 No=1 NS=0 | 1 | 0 | 0 | 0 | 1 | 1 | 1 | 1 |
| Has the ‘standard’ treatment that the control group received been clearly outlined? | Y | Y | Y | N | Y | Y | Y | Y |
| Yes=1 No=0 | 1 | 1 | 1 | 0 | 1 | 1 | 1 | 1 |
| Adequacy of randomisation technique? | Unclear | Unclear | Adequate | Adequate | Adequate | Adequate | Adequate | Adequate |
| Adequate=1 Inadequate/unclear=0 | 0 | 0 | 1 | 1 | 1 | 1 | 1 | 1 |
| Blinding of investigators | N | NS | NS | N | N | N | N | N |
| Yes=1 No/NS=0 | 0 | 0 | 0 | 0 | 0 | 0 | 0 | 0 |
| Blinding of participants | NS | NS | NS | N | N | N | N | NS |
| Yes=1 No/NS=0 | 0 | 0 | 0 | 0 | 0 | 0 | 0 | 0 |
| Blinding of outcome assessor | NS | NS | Y | NS | Y | Y | NS | Y |
| Yes=1 No/NS=0 | 0 | 0 | 1 | 0 | 1 | 1 | 0 | 1 |
| Blinding of data analysis | NS | NS | NS | NS | Y | Y | NS | Y |
| Yes=1 No/NS=0 | 0 | 0 | 0 | 0 | 1 | 1 | 0 | 1 |
| Intention to treat analysis | Y | NS | Y | NS but confirmed | Y | Y | NS | Y |
| Yes=1; Not stated but confirmed on study assessment=1; Stated but not confirmed on study assessment=0; No/NS=0 | 1 | 0 | 1 | 1 | 1 | 1 | 0 | 1 |
| Completion of follow-up (at 6 months if possible) | 84% | 100% | 100% | 100% | 100% | 97.4% | 91.7% | 97.8% |
| 100%=2; >=95%=1; <95%=0; No/NS=0 | 0 | 2 | 2 | 2 | 2 | 1 | 0 | 1 |
| Reasons given when patients were excluded from enrolment, allocation follow-up or analysis? | Y | Y | Y | N | Y | Y | N | Y |
| Yes=1 No=0 | 1 | 1 | 1 | 0 | 1 | 1 | 0 | 1 |
| Has the first author published 2+ RCTs that have been included in this review? | N | N | Y | N | N | N | N | N |
| Yes=0 No=2 | 2 | 2 | 0 | 2 | 2 | 2 | 2 | 2 |
|  |  |  |  |  |  |  |  |  |
| TOTAL SCORE | **9** | **9** | **11** | **8** | **15** | **14** | **8** | **14** |

NS = Not stated, Y=Yes, N=No
